# RIPK3 promotes cell death and NLRP3 inflammasome activation in the absence of MLKL

**DOI:** 10.1038/ncomms7282

**Published:** 2015-02-18

**Authors:** Kate E. Lawlor, Nufail Khan, Alison Mildenhall, Motti Gerlic, Ben A. Croker, Akshay A. D’Cruz, Cathrine Hall, Sukhdeep Kaur Spall, Holly Anderton, Seth L. Masters, Maryam Rashidi, Ian P. Wicks, Warren S. Alexander, Yasuhiro Mitsuuchi, Christopher A. Benetatos, Stephen M. Condon, W. Wei-Lynn Wong, John Silke, David L. Vaux, James E. Vince

**Affiliations:** 1 Inflammation Division, The Walter and Eliza Hall Institute of Medical Research, 1G Royal Parade, Parkville, Victoria 3052, Australia; 2 Department of Medical Biology, the University of Melbourne, Parkville, Victoria 3050, Australia; 3 Department of Clinical Microbiology and Immunology, Sackler Faculty of Medicine, Tel Aviv University, Tel Aviv 69978, Israel; 4 Division of Hematology/Oncology, Boston Children’s Hospital, Harvard Medical School, Boston, Massachusetts 02115, USA; 5 TetraLogic Pharmaceuticals Corporation, 343 Phoenixville Pike, Malvern, Pennsylvania 19355, USA; 6 Institute of Experimental Immunology, University of Zürich, Zürich 8057, Switzerland

## Abstract

RIPK3 and its substrate MLKL are essential for necroptosis, a lytic cell death proposed to cause inflammation via the release of intracellular molecules. Whether and how RIPK3 might drive inflammation in a manner independent of MLKL and cell lysis remains unclear. Here we show that following LPS treatment, or LPS-induced necroptosis, the TLR adaptor protein TRIF and inhibitor of apoptosis proteins (IAPs: X-linked IAP, cellular IAP1 and IAP2) regulate RIPK3 and MLKL ubiquitylation. Hence, when IAPs are absent, LPS triggers RIPK3 to activate caspase-8, promoting apoptosis and NLRP3–caspase-1 activation, independent of RIPK3 kinase activity and MLKL. In contrast, in the absence of both IAPs and caspase-8, RIPK3 kinase activity and MLKL are essential for TLR-induced NLRP3 activation. Consistent with *in vitro* experiments, interleukin-1 (IL-1)-dependent autoantibody-mediated arthritis is exacerbated in mice lacking IAPs, and is reduced by deletion of RIPK3, but not MLKL. Therefore RIPK3 can promote NLRP3 inflammasome and IL-1β inflammatory responses independent of MLKL and necroptotic cell death.

## Abstract (web summary)

RIPK3 can cause necroptotic cell death via MLKL phosphorylation, and activate NLRP3 inflammasome. Here the authors show that MLKL is dispensable for NLRP3 activation by RIPK3, and highlight how different IAP proteins limit RIPK3 induced apoptosis, necroptosis and IL-1 secretion.

## Graphical abstract

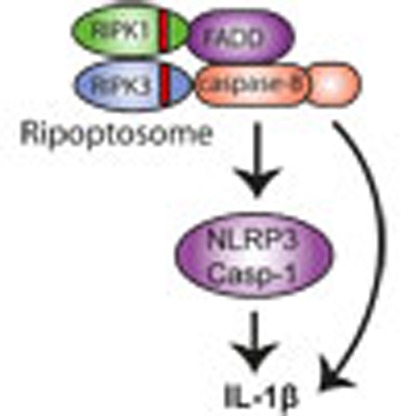

The mammalian inhibitor of apoptosis (IAP) proteins, X-linked IAP (XIAP), cellular IAP1 and IAP2 (cIAP1 and cIAP2) are RING domain E3 ubiquitin ligases^1^. XIAP binds and directly inhibits apoptotic caspase activity (caspase-3, -7 and -9). In contrast, cIAP1/2 indirectly protect from caspase-8-mediated cell death on toll-like receptor (TLR) and death receptor ligation. For example, upon binding of tumour-necrosis factor (TNF) to tumour-necrosis factor receptor 1 (TNFR1), cIAP1/2 ubiquitylate receptor interacting protein kinase-1 (RIPK1)^2^,^3^,^4^ and recruit the linear ubiquitin chain assembly complex (LUBAC)^5^. Ubiquitylated RIPK1 and LUBAC activity propagate pro-survival NF-κB signals, while ubiquitylation of RIPK1 also prevents its association with a FADD-caspase-8 complex that would initiate apoptotic cell death. In circumstances where caspase-8 activity is low and TNF or TLR pathways are activated, cIAP1/2 also repress programmed necrosis, known as necroptosis^6^. Necroptotic signalling requires RIPK1, RIPK3 (refs 7, 8, 9) and the RIPK3 substrate, mixed lineage kinase domain-like (MLKL)^10^,^11^,^12^. On phosphorylation by RIPK3, MLKL has been reported to interact with lipids in the plasma membrane to induce necroptosis^13^,^14^,^15^,^16^.

Recent studies have proposed that cIAP1/2 and XIAP have overlapping roles in the regulation of death receptors, innate pattern recognition receptors and organism development. Combined loss of XIAP and cIAP1, or cIAP1 and cIAP2, causes embryonic lethality at E10.5 with a similar phenotype, and both doubly deficient IAP embryos are rescued to ~E14.5–E16.5 by RIPK1 co-deletion^17^. Similarly, both XIAP and cIAP1/2 have been reported to ubiquitylate RIPK2 to promote anti-microbial cytokine responses following NOD receptor ligation^18^,^19^. Combined loss of XIAP and cIAP1/2 also enhances spontaneous formation of the ripoptosome, a death signalling complex comprised of RIPK1, FADD, caspase-8 and cFLIP^20^,^21^.

We have recently shown that addition of lipopolysaccharide (LPS) or TNF to cells lacking all three IAPs, due to genetic deletion or treatment with IAP antagonist compounds, promotes ripoptosome formation and secretion of the potent pro-inflammatory cytokine interleukin-1β (IL-1β), both *in vitro*^22^, and *in vivo*^23^. TLR stimulation induces the production of inactive precursor IL-1β (pro-IL-1β), which is cleaved following a second stimulus that promotes NOD-like receptor (NLR) inflammasome and associated caspase-1 activity^24^. In macrophages, we found that LPS priming and IAP loss promoted RIPK3-dependent caspase-8 activation leading to caspase-8 cleavage of pro-IL-1β. We also demonstrated that RIPK3 specifically activates the NLRP3–caspase-1 inflammasome^22^. Similarly, it was recently reported that LPS stimulation of caspase-8-deficient dendritic cells induces RIPK3-dependent activation of NLRP3, triggering IL-1β-dependent endotoxic shock *in vivo*^25^. Despite these advances, it remains unclear how IAPs repress LPS-induced RIPK3 activity, how RIPK3 couples to the NLRP3 inflammasome, and whether RIPK3 can induce inflammation directly or can only do so indirectly by induction of necroptosis. We now show that in the absence of IAPs, TLR-induced RIPK3 promotes caspase-8 to activate the NLRP3 inflammasome in the absence of RIPK3 kinase activity and the necroptotic effector MLKL. Furthermore, inflammatory arthritis disease persistence and ankle joint secretion of IL-1β requires RIPK3 and caspase-8, but not MLKL. Therefore, RIPK3 can drive inflammation in the absence of necroptotic cell death.

## Results

### XIAP loss is required for LPS or TNF-induced IL-1β secretion

**Figure 1:**
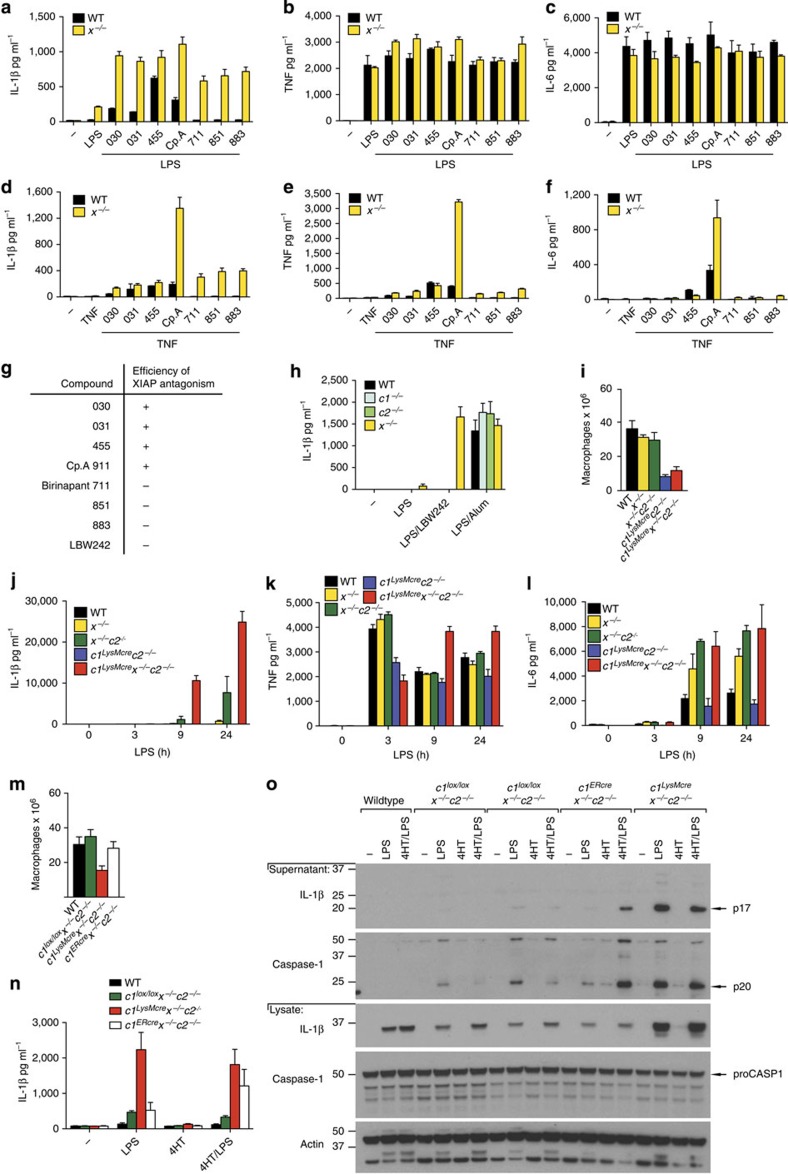
XIAP is required to repress LPS- and TNF-induced IL-1β secretion. (**a**–**f**) WT and *Xiap*-deficient (*x*^−/−^) macrophages were pre-incubated with or without (**a**–**c**) LPS (20 ng ml^−1^) or (**d**–**f**) human Fc-TNF (100 ng ml^−1^) for 2–3 h and cultured with or without IAP antagonists of differing IAP specificities (see **g**). After 24 h, cell supernatants were assayed for (**a**,**d**) IL-1β, (**b**,**e**) TNF and (**c**,**f**) IL-6 levels by ELISA. *n*=3 mice; Data are represented as mean+s.e.m., from one of three experiments. (**g**) Efficiency of functional XIAP antagonism by IAP antagonist compounds (+, high; −,low). (**h**) WT, *cIAP1*^−/−^ (*c1*^−/−^), *cIAP2*^−/−^ (*c2*^−/−^) and *Xiap*^−/−^ (*x*^−/−^) BMDM were primed with LPS (20 ng ml^−1^) for 3 h and cultured with the IAP antagonist LBW242 (20 μM) or alum (320 μg ml^−1^) for a further 6 h. Secreted IL-1β was measured in supernatants by ELISA. *n*=3 mice; mean+s.e.m. (**i**) Yield of macrophages from WT and IAP mutant bone marrow after 6 days of culture with L929 cell conditioned media. *n*=3–6 mice per genotype, mean+s.e.m. (**j**–**l**) WT and IAP mutant macrophages were stimulated with LPS (20 ng ml^−1^) for up to 24 h, and (**j**) IL-1β, (**k**) TNF and (**l**) IL-6 levels were assayed in supernatants by ELISA. *n*=3–4 mice, data are represented as mean+s.e.m., one of three experiments. (**m**) Yield of WT, *c1*^*lox/lox*^*x*^−/−^*c2*^−/−^, *c1*^*LysMcre*^*x*^−/−^*c2*^−/−^ and *c1*^*ERcre*^*x*^−/−^*c2*^−/−^ bone marrow macrophages after 6 days of culture with L929 cell conditioned media. *n*=3–6 mice per genotype, mean+s.e.m. (**n**,**o**) WT, *c1*^*lox/lox*^*x*^−/−^*c2*^−/−^, *c1*^*LysMcre*^*x*^−/−^*c2*^−/−^ and *c1*^*ERcre*^*x*^−/−^*c2*^−/−^ macrophages were pulsed for 16 h with 4′-hydroxy-tamoxifen (4HT 1000, nM) and then rested for 10 h prior to stimulation with or without LPS (50 ng ml^−1^) for a further 8 h. (**n**) Secreted IL-1β was measured in supernatants by ELISA, *n*=3 mice per group; c1^LysMcre^x^−/−^c2^−/−^ (*n*=2), mean+s.d., one of three experiments, and (**o**) IL-1β and caspase-1 activation assayed by immunoblot of supernatants and lysates. Representative blot from the analysis of 4 *c1*^*ERcre*^*x*^−/−^*c2*^−/−^ mice. Full-size immunoblots are presented in Supplementary Fig. 9.

IAP antagonist compounds are better tolerated *in vivo* when their functional affinity for XIAP is less than for cIAP1/2 ref. 26. We therefore tested a range of IAP antagonists with varying IAP specificities^26^ to assess whether XIAP antagonism might contribute to toxicity by inducing macrophage secretion of pro-inflammatory cytokines, such as IL-1β (Fig. 1a–h). Only bivalent IAP antagonists ‘termed Smac-mimetics’, which antagonized XIAP efficiently, in addition to cIAP1/2 (030, 031, 455, Cp.A^26^; Fig. 1g), caused significant IL-1β secretion in LPS- or TNF-primed wild-type (WT) bone marrow-derived macrophages (BMDM) (Fig. 1a,d). In contrast, cIAP1/2-selective IAP antagonists (711 (birinapant), 851, 883, LBW242) only promoted IL-1β secretion in *Xiap*^−/−^ (*x*^−/−^) cells (Fig. 1a,d,h). LPS or TNF priming followed by IAP antagonist addition did not alter TNF or IL-6 secretion to the same extent (Fig. 1b,c,e,f).

To validate the results seen using Smac-mimetic compounds, we examined mice lacking genes for one or more IAPs (Fig. 1h–o and Supplementary Fig. 1a–d). Significantly fewer *cIAP1*^*fl/fl.LysMcre*^*cIAP2*^−/−^ (*c1*^*LysMcre*^*c2*^−/−^) and *cIAP1*^*fl/fl.LysMcre*^*Xiap*^−/−^*cIAP2*^−/−^ (*c1*^*LysMcre*^*x*^−/−^*c2*^−/−^) BMDM were recovered compared with the yield from WT, *Xiap*^−/−^ (*x*^−/−^) or *Xiap*^−/−^*cIAP2*^−/−^ (*x*^−/−^*c2*^−/−^) BM (Fig. 1i). Despite this, and as previously reported by us^22^, when all three IAPs were deleted we observed maximal secretion of the inflammasome dependent cytokines IL-1β and IL-18 in BMDM stimulated with LPS or TNF, and the levels were considerably greater than when IAPs were antagonized by the Smac-mimetic Cp.A (Fig. 1j and Supplementary Fig. 1a–d). Prolonged incubation with LPS also caused IL-1β secretion in *x*^−/−^ BMDM (that was enhanced by co-deletion of cIAP2), although levels produced after 9 h were ~70-fold lower when compared with IAP triple knockout macrophages (Fig. 1j and Supplementary Fig. 1a–d). In contrast, co-deletion of cIAP1 and cIAP2 did not induce IL-1β activation in primed cells (Fig. 1j and Supplementary Fig. 1c,d). LPS-induced TNF and IL-6 secretion were similar in WT and XIAP-deficient BMDM, while *c1*^*LysMcre*^*x*^−/−^*c2*^−/−^ macrophages secreted more TNF at later time points (Fig. 1k). Compared with LPS, TNF-induced less cytokine secretion, but did cause a late increase in TNF secretion in *c1*^*LysMcre*^*c2*^−/−^ BMDM that was greater than in WT, *x*^−/−^ and *x*^−/−^*c2*^−/−^ cells (Supplementary Fig. 1c,d).

To ensure the markedly enhanced IL-1β secretion observed in LPS- or TNF-stimulated *c1*^*LysMcre*^*x*^−/−^*c2*^−/−^ BMDM did not reflect cell intrinsic defects in macrophage differentiation, we generated BMDM from *c1*^*fl/fl ERcre*^*x*^−/−^*c2*^−/−^ (*c1*^*ERcre*^*x*^−/−^*c2*^−/−^) mice. Unlike *c1*^*LysMcre*^*x*^−/−^*c2*^−/−^ mice, the number of macrophages recovered from *c1*^*ERcre*^*x*^−/−^*c2*^−/−^ BM was normal (Fig. 1m). Treatment of *c1*^*ERcre*^*x*^−/−^*c2*^−/−^ BMDM for 42–48 h with 4-hydroxy-tamoxifen (4HT) efficiently deleted cIAP1, and caused significant cell death that correlated with increased caspase-8 processing (Supplementary Fig. 1e,f). In contrast, 4HT treatment of *c1*^*ERcre*^*x*^−/−^*c2*^−/−^ BMDM for 24 h did not cause cell death but reduced cIAP1 protein levels to that observed in *c1*^*LysMcre*^*x*^−/−^*c2*^−/−^ cells (~50% of WT; Supplementary Fig. 1e,f). Under these conditions, LPS stimulation of 4HT-treated *c1*^*ERcre*^*x*^−/−^*c2*^−/−^ BMDM mimicked *c1*^*LysMcre*^*x*^−/−^*c2*^−/−^ BMDM responses, and induced increased levels of active IL-1β and caspase-1 (Fig. 1n,o). Therefore, the removal of all three IAP proteins promotes maximal TNF- or LPS-induced IL-1β secretion, but the inhibition or deletion of XIAP is essential.

### XIAP limits TLR- and TNF-induced apoptosis and necroptosis

IL-1β secretion caused by LPS and Cp.A treatment occurs prior to a loss of macrophage plasma membrane integrity, but these cells eventually die^22^. Since XIAP critically prevents IL-1β activation following addition of LPS, we tested whether it also reduced apoptotic or necroptotic cell death.

**Figure 2:**
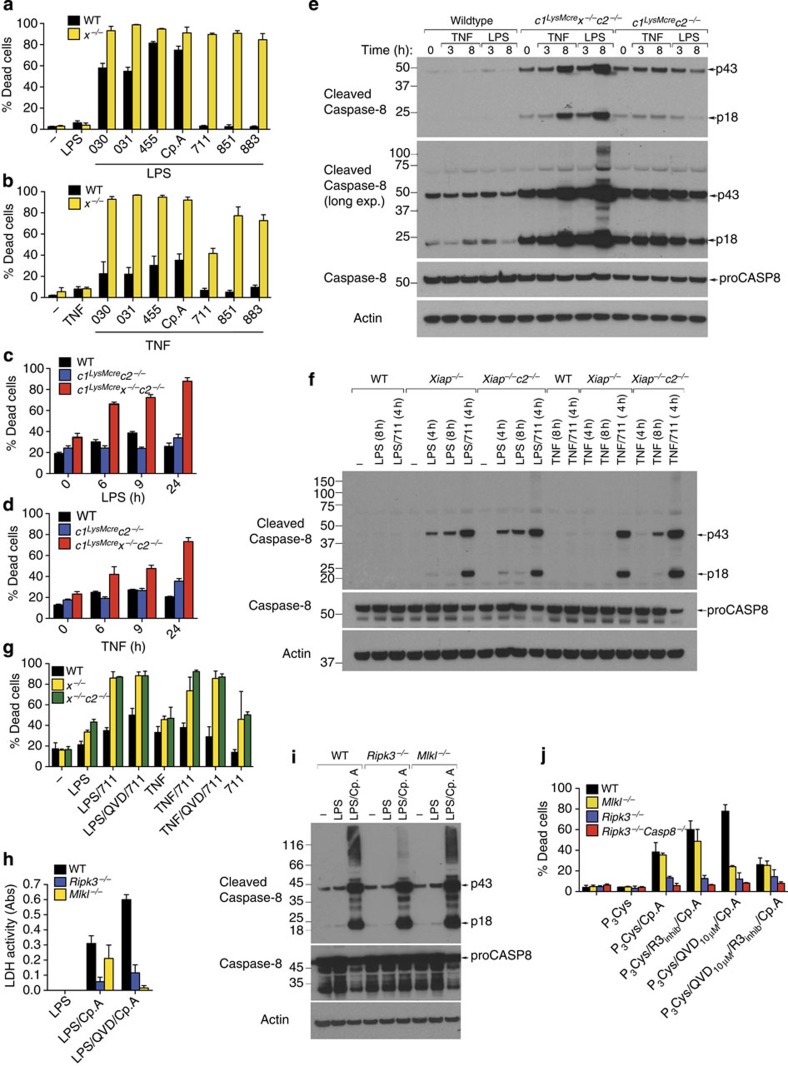
XIAP limits LPS- and TNF-induced apoptosis and necroptosis in macrophages. (**a**,**b**) WT and *x*^−/−^ BMDM were pre-incubated with or without (**a**) LPS (20 ng ml^−1^) or (**b**) TNF (100 ng ml^−1^) for 2–3 h and were cultured with IAP antagonists of differing IAP specificities (500 nM; see Fig. 1g) as indicated for 24 h. Cell death was assessed by flow cytometric analysis of PI uptake. Data are presented as the % Dead cells, *n*=3 mice, mean+s.e.m., one of two experiments. (**c**,**d**) WT, *c1*^*LysMcre*^*c2*^−/−^ or *c1*^*LysMcre*^*x*^−/−^*c2*^−/−^ BMDM were stimulated with (**c**) LPS (20 ng ml^−1^) or (**d**) TNF (100 ng ml^−1^) and cell death (% Dead cells) measured by flow cytometric analysis of PI uptake. *n*=3 mice, mean+s.e.m., one of three experiments. (**e**) WT, *c1*^*LysMcre*^*c2*^−/−^ or *c1*^*LysMcre*^*x*^−/−^*c2*^−/−^ BMDM were stimulated with LPS (20 ng ml^−1^) or TNF (100 ng ml^−1^) and lysates were analyzed for caspase-8 processing by immunoblot as indicated. Representative of one of two experiments. Full-size immunoblots are presented in Supplementary Fig. 10. (**f**) WT, *x*^−/−^ and *x*^−/−^*c2*^−/−^ BMDM were primed with LPS (20 ng ml^−1^) or TNF (100 ng ml^−1^) and cultured with cIAP1/2-selective antagonist, 711 (500 nM), as indicated, and lysates analyzed for caspase-8 processing by immunoblot. Representative of one of three experiments. Full-size immunoblots are presented in Supplementary Fig. 10. (**g**) WT, *x*^−/−^, or *x*^−/−^*c2*^−/−^ BMDM were primed for 3 h with LPS (20 ng ml^−1^) or TNF (100 ng ml^−1^), and as indicated cultured with the cIAP1/2-selective antagonist, 711 (500 nM), in the presence or absence of Q-VD-OPh (20 μM, added in the last 20 min of priming). Cell death was measured after 24 h by PI uptake. *n*=3 mice, mean+s.e.m., one of two experiments. (**h**) WT, *Mlkl*^−/−^ and *Ripk3*^−/−^ BMDM were primed for 3 h with LPS and treated with Q-VD-OPh (20 μM) as indicated for the final 20 min prior to addition of Cp.A (500 nM). Cell death was measured by assaying lactate dehydrogenase (LDH) release (*n*=3 mice per genotype). (**i**) Cell lysates of WT, *Mlkl*^−/−^ and *Ripk3*^−/−^ BMDM primed with LPS for 3 h and treated with Cp.A (500 nM) for 6 h were analyzed by immunoblot. Representative immunoblot analysis of three mice of each genotype. Full-size immunoblots are presented in Supplementary Fig. 10. (**j**) WT, *Ripk3*^−/−^, *Mlkl*^−/−^ and *Ripk3*^−/−^*Caspase-8*^−/−^ BMDM were primed with Pam_3_Cys (2.5 μg ml^−1^) for 3 h, treated with Q-VD-OPh in the final 20 min of priming, and Cp.A added, as specified, for 24 h. In some cases RIP3 kinase inhibitor (R3 inhib, GSK872; 1 μM) was added 20 min prior to the addition of Cp.A. Cell death was measured by PI uptake and flow cytometric analysis (% Dead cells). *n*=3 mice, mean+s.e.m.

Inhibiting or removing cIAP1 alone, or in combination with cIAP2, sensitizes many cell types to death receptor triggered caspase-8 activation and apoptosis^3^,^4^,^27^. Consistent with this, cIAP1/2 targeting by 711 (birinapant) and TNF stimulation induced significant apoptotic or necroptotic cell death of immortalized murine dermal fibroblasts (Supplementary Fig. 1g). Surprisingly, BMDM were more resistant to cell death induced by cIAP1/2 targeted IAP antagonists and TNF or LPS stimulation (Fig. 2a,b), as were *c1*^*LysMcre*^*c2*^−/−^ macrophages (Fig. 2c,d). In contrast, BMDM were susceptible to apoptosis induced by LPS and TNF stimulation when XIAP was co-deleted with cIAP1/2, or all three IAPs were targeted by IAP antagonists (Fig. 2a–d).

XIAP directly inhibits effector caspase activity. Yet, immunoblots revealed that the processing of the initiator caspase, caspase-8, was increased following LPS or TNF stimulation of BMDM lacking XIAP, and particularly when all three IAPs were deleted or inhibited (Fig. 2e,f and Supplementary Fig. 1f,h). In contrast, cIAP1/2 depletion alone had less impact on LPS or TNF-induced caspase-8 activation (Fig. 2e,f and Supplementary Fig. 1h). These data identify XIAP as an important repressor of both TNFR1- and TLR-induced caspase-8 activation and apoptosis.

To examine the relative contributions of cIAP1/2 and XIAP to necroptotic cell death, IAP gene-targeted or IAP antagonist-treated BMDM were co-treated with the caspase inhibitor (Q-VD-Oph) in combination with either LPS or TNF; conditions previously shown to induce RIPK3–MLKL-dependent necroptosis^7^,^20^,^28^. We observed that LPS- and TNF-induced necroptotic killing of BMDM was significantly enhanced in XIAP deleted cells (Fig. 2g), as well as IL-1β production (Supplementary Fig. 1i). This was most striking for TNF-induced necroptosis, where in combination with the compound 711 and caspase inhibition (Q-VD-OPh), TNF failed to induce necroptosis of WT BMDM, but efficiently killed XIAP-deficient BMDM (Fig. 2g). Interestingly, RIPK3 deletion not only abrogated necroptotic cell death, but also significantly delayed and diminished apoptotic cell death induced by LPS and Cp.A, when compared with WT and *Mlkl*^−/−^ BMDM (Fig. 2h and Supplementary Fig. 2a,b). Delayed apoptotic death in *Ripk3*^−/−^, but not *Mlkl*^−/−^, BMDM correlated with reduced caspase-8 modification, possibly ubiquitylation, that is linked to enhanced caspase-8 function (Fig. 2i)^29^. Subsequently, complete abrogation of TLR and Cp.A-induced apoptosis was observed in *Ripk3*^−/−^*Casp8*^−/−^ macrophages (Fig. 2j, Video 1 and Supplementary Fig. 2c,d). In contrast to RIPK3-deficient cells, the loss of MLKL only blocked necroptosis (Fig. 2h–j and Supplementary Fig. 2a,b).

Collectively, these data show that RIPK3 can promote activation of caspase-8 apoptotic and MLKL necroptotic signalling, and XIAP limits both these cell death pathways.

### RIPK3 activates NLRP3 independent of MLKL

**Figure 3:**
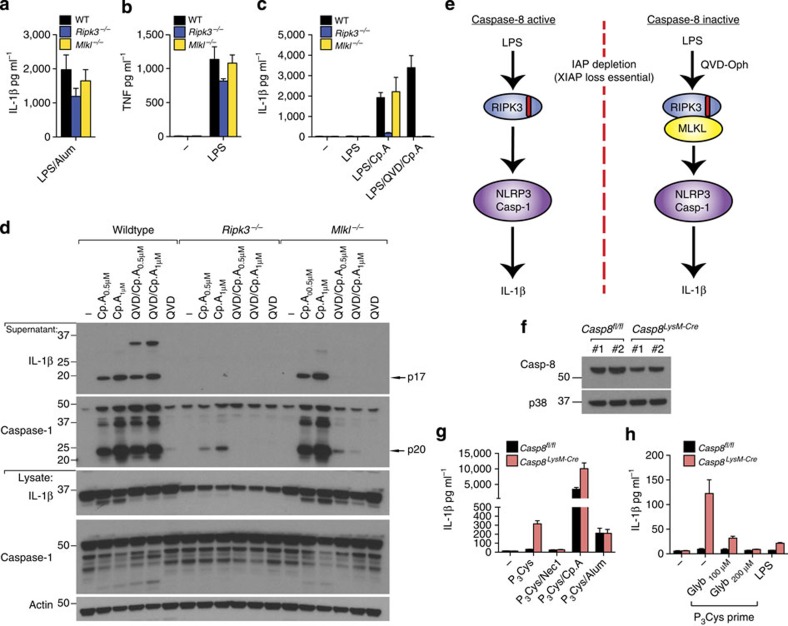
RIPK3 activates caspase-1 independent of MLKL unless caspase-8 is inhibited. (**a**–**c**) WT, *Mlkl*^−/−^ and *Ripk3*^−/−^ BMDM were primed with LPS (20 ng ml^−1^) for 3 h and cultured with Q-VD-OPh (20 μM), where indicated, which was added in the last 20 min of priming. Cells were then stimulated with Cp.A (500 nM) or alum (300 μg ml^−1^) for a further 6 h. Supernatants were analyzed for (**a**,**c**) IL-1β and (**b**) TNF by ELISA. *n*=3 mice per genotype. Data are represented as mean+s.e.m. and are representative of one of three independent experiments. (**d**) WT, *Mlkl*^−/−^ and *Ripk3*^−/−^ BMDM were primed with LPS for 2.5 h. In the last 20 min of priming, cells were incubated with Q-VD-OPh (20 μM) and then cultured with Cp.A (1 μM) for 5 h. Cell supernatants and lysates were analyzed by immunoblot. Representative of one of three experiments. Full-size immunoblots are presented in Supplementary Fig. 11. (**e**) Schematic depicting how RIPK3 signals IL-1β activation based on the data presented in Figs 1, 2, 3. (**f**) Lysates from WT (*Casp8*^*fl/fl*^) littermate and caspase-8-deficient (*Casp8*^*LysMcre*^) BMDM (*n*=2 mice) were subjected to immunoblot to assess efficiency of caspase-8 deletion. Full-size immunoblots are presented in Supplementary Fig. 11. (**g**) WT littermate and *Caspase-8*^LysMcre^ BMDM were primed for 3 h with Pam_3_Cys (2 μg ml^−1^), and as indicated treated with Nec-1 (50 μM) in the last 20 min of priming. Cells were then exposed to Cp.A (500 nM), as specified, for a further 24 h, after which IL-1β release was measured by ELISA. *n*=3 mice per genotype, mean+s.e.m. Representative of one of three experiments. (**h**) WT littermate and *Caspase-8*^LysMcre^ BMDM were pre-incubated with glyburide for 20 min, as indicated, and cultured with Pam_3_Cys (2 μg ml^−1^) or LPS (100 ng ml^−1^) for 24 h. Cell supernatants were assayed for IL-1β by ELISA. *n*=4 mice per genotype, mean+s.e.m. Representative of one of two experiments.

We next sought to understand whether RIPK3–MLKL-mediated necroptotic death signalling is also necessary for RIPK3 to induce NLRP3 inflammasome activation. Examination of responses in LPS-primed WT, *Ripk3*^−/−^ and *Mlkl*^−/−^ macrophages to the NLRP3 activator, alum, revealed similar IL-1β and TNF secretion (Fig. 3a,b). In contrast, and as expected^22^, *Ripk3*^−/−^ BMDM were defective in LPS- and Cp.A-induced NLRP3–caspase-1 and IL-1β activation (Fig. 3c,d). Surprisingly, however, caspase-1 and IL-1β activation in *Mlkl*^−/−^ BMDM was similar to that in WT cells (Fig. 3c,d), demonstrating that RIPK3 can specifically promote NLRP3–caspase-1 and IL-1β activation in the absence of MLKL, and hence in the absence of necroptosis.

We also blocked caspase function to force RIPK3–MLKL activation by treating LPS- and Cp.A-stimulated BMDM with Q-VD-OPh. Strikingly, caspase-1 and IL-1β processing and secretion observed in *Mlkl*^−/−^ or *Ripk3*^−/−^ BMDM stimulated by LPS and Cp.A was abolished when cells were co-treated with a concentration of Q-VD-OPh that inhibits caspase-8 but not caspase-1 (Fig. 3c,d)^22^. In comparison, WT BMDM co-treated with Q-VD-OPh secreted processed IL-1β and caspase-1 at similar, if not higher, levels compared with LPS and Cp.A treatment only (Fig. 3c,d). These results suggest that RIPK3 can promote NLRP3 activation in both an MLKL-independent and -dependent manner, which is dictated by the levels of caspase-8 activity (summarized in Fig. 3e).

To verify that caspase-8 loss promotes IL-1β secretion via RIPK3–MLKL–NLRP3, we deleted caspase-8 in myeloid cells (*Caspase-8*^*LysMcre*^), as *Caspase-8*^−/−^ mice are embryonic lethal. As previously reported^30^, BMDM derived from *Caspase-8*^*LysMcre*^ mice showed inefficient caspase-8 deletion, ~30–50% (Fig. 3f). Nevertheless, Pam_3_Cys (TLR1/2) priming alone resulted in appreciable IL-1β secretion from *Caspase-8*^*LysMcre*^ macrophages, and enhanced Cp.A-mediated IL-1β and TNF secretion (Fig. 3g and Supplementary Fig. 2e). Pam_3_Cys-induced IL-1β secretion in *Caspase-8*^*LysMcre*^ BMDM was inhibited by the RIPK1 kinase inhibitor necrostatin-1 (Nec-1; Fig. 3g) and the NLRP3 inhibitor glyburide (Fig. 3h). Therefore, when caspase-8 function is reduced, RIPK3–MLKL signals NLRP3–caspase-1 activation.

### RIPK3 kinase activity is dispensable for IL-1β activation

**Figure 4:**
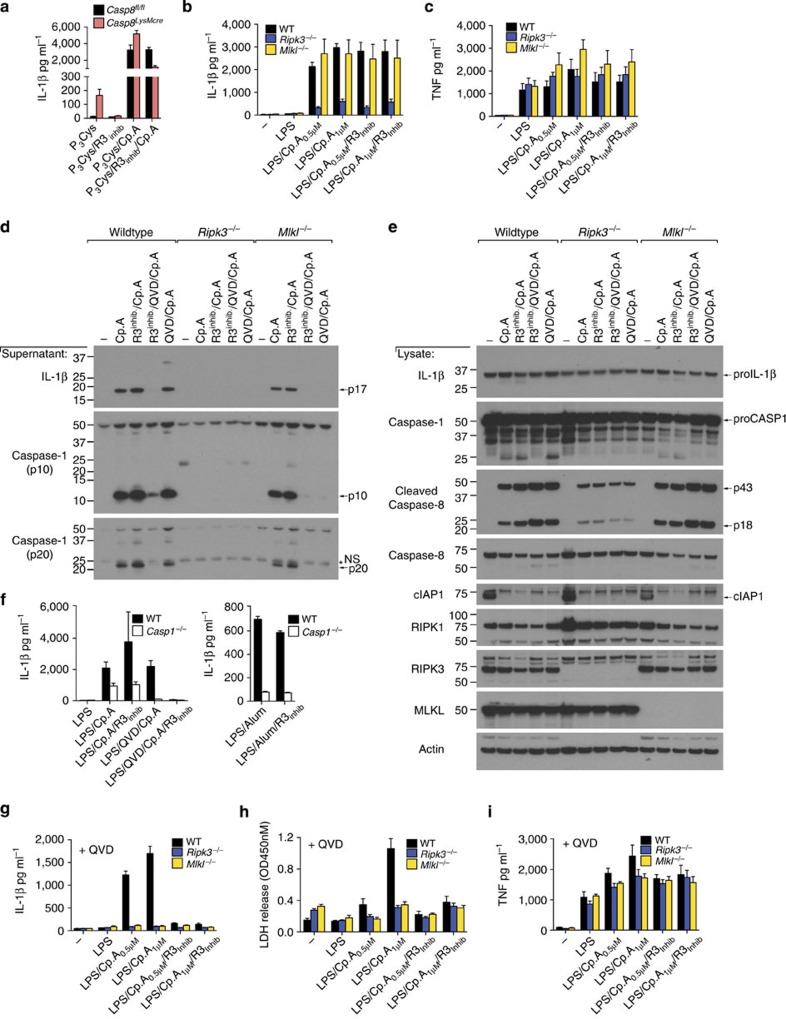
RIPK3 kinase activity is not required for MLKL-independent activation of NLRP3. (**a**) WT littermate and *Caspase-8*^LysMcre^ BMDM were primed for 3 h with Pam_3_Cys (2 μg ml^−1^), and as indicated RIPK3 inhibitor (R3 inhib GSK872; 1 μM) was added in the last 20 min of priming. Cells were then exposed to Cp.A (500 nM), as specified, for a further 24 h. Levels of IL-1β secretion were measured by ELISA. *n*=4 mice per genotype, mean+s.e.m. (**b**,**c**) WT, *Ripk3*^−/−^ and *Mlkl*^−/−^ BMDM were primed for 3 h with LPS (20 ng ml^−1^) in the absence or presence of RIPK3 inhibitor (R3 inhib; 1 μM), prior to addition of Cp.A for a further 6 h. Supernatants were assayed for (**b**) IL-1β and (**c**) TNF by ELISA or death assessed by lactate dehydrogenase (LDH) activity (see Supplementary Fig. 2f). *n*=3 mice per genotype, mean+s.e.m. (**d**,**e**) Cell supernatants (**d**) and lysates (**e**) from WT, *Mlkl*^−/−^ and *Ripk3*^−/−^ BMDM primed with LPS (3 h) and treated with Q-VD-OPh (20 μM) and R3 inhibitor (1 μM, last 20 min of priming), as indicated, and subsequently treated with Cp.A (1 μM, 5 h) were analyzed by immunoblot as indicated. One of three experiments. Full-size immunoblots are presented in Supplementary Fig. 12. (**f**) WT and *caspase-1*^−/−^ BMDM were primed for 3 h with LPS, and as indicated treated with Q-VD-OPh (20 μM) and R3 inhib (1 μM) for the last 20 min of priming. BMDM were then cultured with Cp.A (1 μM) or Alum (300 μg ml^−1^) for a further 6 h. Culture supernatants were assayed for IL-1β levels by ELISA. *n*=3 mice, mean+s.e.m., one of two experiments. (**g**–**i**) WT, *Ripk3*^−/−^ and *Mlkl*^−/−^ BMDM were primed for 3 h with LPS (20 ng ml^−1^) in the presence of Q-VD-OPh (20 μM), and where indicated 1 μM RIPK3 inhibitor (R3 inhib), prior to addition of Cp.A for a further 6 h. Supernatants were assayed for (**g**) IL-1β and (**i**) TNF by ELISA, and (**h**) cell death was measured via an LDH assay. *n*=3 mice per genotype, mean+s.e.m. *NS, non-specific band.

To test if the kinase activity of RIPK3 is necessary for MLKL-independent NLRP3 activation, we utilized the RIPK3 kinase inhibitor GSK872 (ref. 31). Spontaneous IL-1β secretion from Pam_3_Cys-treated *Caspase-8*^*LysMcre*^ BMDM was prevented by RIPK3 kinase inhibition (Fig. 4a). In contrast, RIPK3 kinase inhibition did not alter caspase-1 and IL-1β activation, or TNF secretion, induced by LPS and Cp.A stimulation of WT, *Ripk3*^−/−^ or *Mlkl*^−/−^ BMDM (Fig. 4b–e). Likewise, RIPK3 kinase inhibition did not affect TLR and Cp.A-triggered caspase-8 activation and apoptosis (Figs 2j,4e and Supplementary Fig. 2f), nor the caspase-8-dependent processing of IL-1β observed in caspase-1-deficient macrophages (Fig. 4f)^22^. Therefore, RIPK3 kinase activity is not required for RIPK3-mediated caspase-8 activation, or caspase-8-mediated IL-1β maturation and secretion.

In contrast, similar to the deletion of caspase-8, when RIPK3–MLKL-mediated inflammasome activation was forced by Q-VD-OPh treatment of LPS- and Cp.A-stimulated macrophages, RIPK3 kinase activity was essential for caspase-1 and IL-1β processing and secretion (Fig. 4d,g and Supplementary Fig. 2g), as well as necroptosis (Figs 2j,4h). RIPK3 kinase inhibition did not alter TNF secretion under these conditions (Fig. 4i), nor did it impact NLRP3 activation by alum (Fig. 4f).

Collectively, these data demonstrate that RIP3 kinase activity can cause IL-1β secretion via RIPK3–MLKL-dependent NLRP3 activation when caspase-8 function is reduced. However, in the presence of caspase-8, RIPK3 kinase activity is not required to promote NLRP3–caspase-1 activation of IL-1β that can occur in the absence of MLKL.

### In the absence of MLKL, RIPK3/caspase-8 activate NLRP3

**Figure 5:**
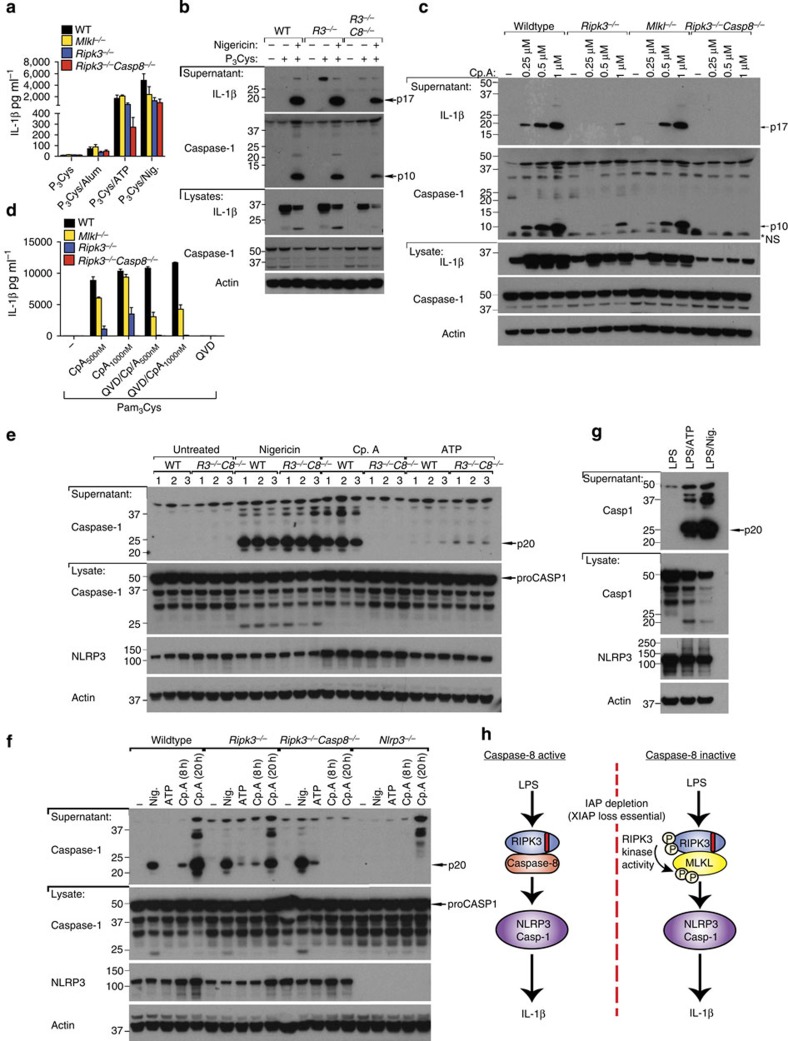
Deletion of both RIPK3 and caspase-8 abrogates TLR- and Cp.A-induced activation of caspase-1 and IL-1β. (**a**) WT, *Mlkl*^−/−^, *Ripk3*^−/−^ and *Ripk3*^−/−^*Caspase-8*^−/−^ BMDM were primed with Pam_3_Cys for 3 h and incubated with Alum (300 μg ml^−1^) for 6 h, and ATP (5 mM) or nigericin (10 μM) for 40 min. Supernatants were assayed for IL-1β release. *n*=3 mice per genotype, mean+s.e.m., one of three experiments. (**b**) WT, *Ripk3*^−/−^ and *Ripk3*^−/−^*Caspase-8*^−/−^ BMDM were primed with Pam_3_Cys for 3 h and cultured with Nigericin as indicated for 40 min. Supernatants and lysates were analyzed by immunoblot. One of two experiments. Full-size immunoblots are presented in Supplementary Fig. 13. (**c**) WT, *Mlkl*^−/−^, *Ripk3*^−/−^ and *Ripk3*^−/−^*Caspase-8*^−/−^ BMDM were primed with Pam_3_Cys for 3 h and incubated with increasing concentrations of Cp.A for 24 h and supernatants and lysates were analyzed by immunoblot. One of two experiments. Full-size immunoblots are presented in Supplementary Fig. 13. (**d**) WT, *Mlkl*^−/−^, *Ripk3*^−/−^ and *Ripk3*^−/−^*Caspase-8*^−/−^ BMDM were primed with Pam_3_Cys for 3 h, and as indicated Q-VD-OPh (20 μM) for the last 20 min of priming, and then cells were treated where shown with Cp.A for a further 6 h. Supernatants were assayed for IL-1β levels. *n*=3 mice per genotype; mean+s.e.m., representative of one of three experiments. (**e**) Unprimed WT and *Ripk3*^−/−^*Caspase-8*^−/−^ BMDM were cultured with Nigericin (10 μM, 2 h), Cp.A (1 μM, 20 h) and ATP (5 mM, 2 h), and supernatants and lysates were analyzed by immunoblot for caspase-1 activation and NLRP3 levels. *n*=3 mice per genotype (numbered). Full-size immunoblots are presented in Supplementary Fig. 13. (**f**) Unprimed WT, *Ripk3*^−/−^, *Ripk3*^−/−^*Caspase-8*^−/−^ and *Nlrp3*^−/−^ BMDM were stimulated with Nigericin (10 μM, 2 h), Cp.A (1 μM), and ATP (5 mM, 2 h) as indicated, and supernatants and cell lysates were analyzed by immunoblot. One of three experiments. Full-size immunoblots are presented in Supplementary Fig. 13. (**g**) WT BMDM were primed with LPS (20 ng ml^−1^) for 3 h, stimulated with Nigericin or ATP, and lysates analyzed by immunoblot. (**h**) Schematic depicting how RIPK3 signals NLRP3–caspase-1 and IL-1β activation based on the data presented in Figs 1, 2, 3, 4, 5. *NS, non-specific band.

Our data suggest that caspase-8 may engage NLRP3-associated caspase-1 to activate IL-1β. In view of the incomplete caspase-8 deletion in *Caspase-8*^*LysMcre*^ mice, we tested this hypothesis by examining *Ripk3*^−/−^*Caspase-8*^−/−^ macrophages. We, and others, have recently reported that *Ripk3*^−/−^*Caspase-8*^−/−^ BMDM are defective in TLR-induced inflammasome priming^32^,^33^. Despite this, Pam_3_Cys induced sufficient inflammasome priming to allow studies into inflammasome activation, where we observed significant caspase-1 and IL-1β activation in *Ripk3*^−/−^*Caspase-8*^−/−^ BMDM in response to the NLRP3 stimuli ATP or nigericin (Fig. 5a,b). In contrast, no caspase-1 or IL-1β activation, nor cell death, was detectable in either LPS or Pam_3_Cys-primed *Ripk3*^−/−^*Caspase-8*^−/−^ BMDM treated with Cp.A for 6–24 h, compared with the significant accumulation observed over time in WT, *Ripk3*^−/−^ and *Mlkl*^−/−^ BMDM (Figs 2j,5c,d, Supplementary Fig. 3 and Video 1). These results mirrored our findings in *Ripk3*^−/−^ BMDM treated with Q-VD-OPh (to inhibit caspase-8) and Cp.A (Fig. 5d, Fig. 2j and Supplementary Fig. 3).

To circumvent the priming defects of *Ripk3*^−/−^*Caspase-8*^−/−^ BMDM, we attempted to activate NLRP3 using unprimed cells. Notably, basal levels of NLRP3 expression in *Ripk3*^−/−^*Caspase-8*^−/−^ BMDM were comparable to WT and *Ripk3*^−/−^ cells (Fig. 5e,f). Nigericin treatment induced significant NLRP3-dependent caspase-1 processing and secretion in unprimed BMDM, which was comparable in WT and *Ripk3*^−/−^*Caspase-8*^−/−^ BMDM (Fig. 5e,f). For reasons unclear, unprimed cells only responded weakly to ATP treatment, despite robust ATP-mediated caspase-1 activation in cells first primed with LPS (Fig. 5e–g). In contrast to nigericin stimulation, Cp.A-mediated NLRP3-dependent caspase-1 processing and secretion was completely absent in *Ripk3*^−/−^*Caspase-8*^−/−^ BMDM when compared with WT BMDM, and the significant caspase-1 processing observed in *Ripk3*^−/−^ BMDM after 20 h treatment (Fig. 5e,f). These findings show that upon IAP loss, RIPK3 and caspase-8 specifically activate NLRP3-associated caspase-1 (summarized in Fig. 5h).

### RIPK1 inhibits RIPK3 activation of the inflammasome

**Figure 6:**
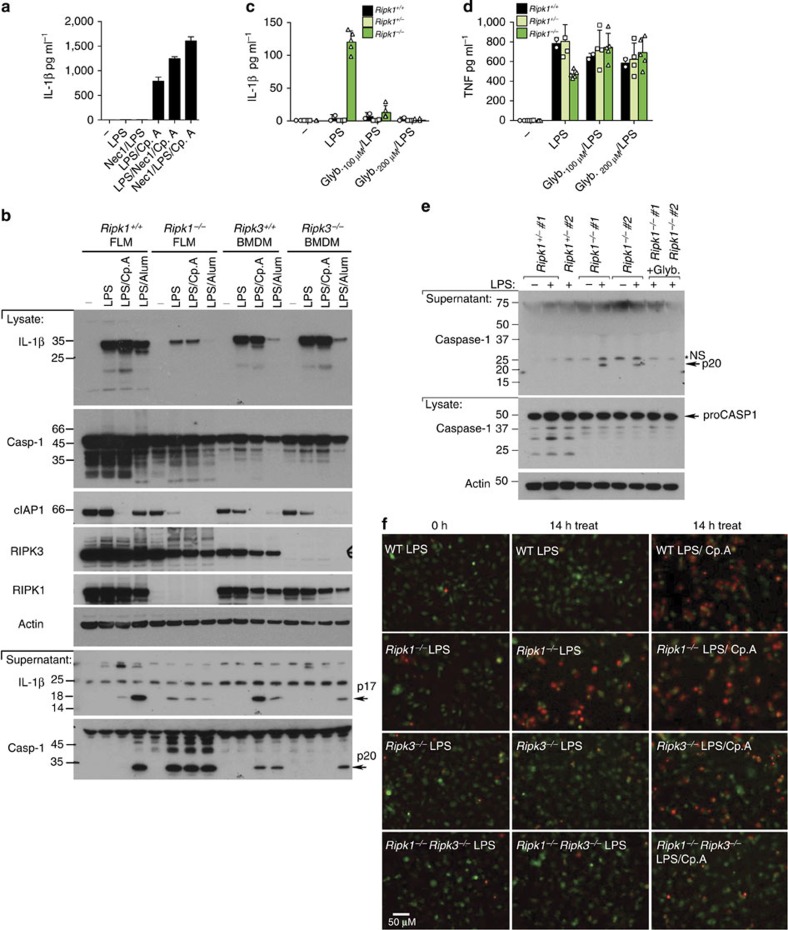
RIPK1 represses LPS-induced RIPK3 activity. (**a**) WT BMDM were either pre-treated for 30 min with Nec-1 (50 μM), or cultured with Nec-1 in the final 30 min of priming with LPS (20 ng ml^−1^) for 3 h. Cp.A (500 nM) was added, as indicated, and cells were cultured for 6 h. Supernatants were assayed for IL-1β. *n*=3 mice per group, mean+s.e.m. One of two experiments. (**b**) WT and *Ripk1*^−/−^ FLM, and WT and *Ripk3*^−/−^ BMDM were primed with LPS for 3 h and stimulated with Cp.A (500 nM) or Alum (300 μg ml^−1^) for a further 6 h, and supernatants and lysates were assayed by immunoblot. Full-size immunoblots are presented in Supplementary Fig. 14. (**c**–**e**) WT, *Ripk1*^+/−^ and *Ripk1*^−/−^ FLDM were treated with glyburide for 20 min and then stimulated with LPS (20 ng ml^−1^) for 6–8 h. Data shows three to five embryos of each genotype. (**c**,**d**) Supernatants were assayed for (**c**) IL-1β and (**d**) TNF production. Data symbols represent individual mice from three experiments. (**e**) Cell lysates and supernatants were blotted for caspase-1 cleavage. *n*=2 individual mice (numbered). Full-size immunoblots are presented in Supplementary Fig. 14. (**f**) WT *Ripk1*^+/+^, *Ripk1*^−/−^, *Ripk3*^−/−^ and *Ripk1*^−/−^*Ripk3*^−/−^ FLDM were labelled with cell tracker green (green) and cultured with LPS (20 ng ml^−1^) for 1 h, prior to stimulation with Cp.A, as indicated, and PI addition (red). Cells were imaged from 2h post-LPS addition every 30 min for 14 h. Supplementary Figure 4g shows additional treatments (LPS/Cp.A/QVD) used in this experiment. Representative images of one of three experiments. *NS, non-specific band.

RIPK1 is often requisite for RIPK3 activation and necroptosis^6^ and therefore may play a role in TLR and Cp.A-induced IL-1β activation. However, we observed that the RIPK1 kinase inhibitor, Nec-1, did not prevent IL-1β secretion in LPS- and Cp.A-treated BMDM (Fig. 6a). This conflicts with the ability of Nec-1 to prevent Pam_3_Cys-induced IL-1β secretion in caspase-8-deficient BMDM (Fig. 3g). However, Pam_3_Cys induces autocrine TNF production to activate RIPK3 in a TNFR1/RIPK1-dependent manner, whereas LPS can directly engage RIPK3 via TRIF^31^.

To further examine if RIPK1 can contribute to NLRP3–caspase-1 activation following LPS stimulation of IAP-depleted cells, we generated *Ripk1*^−/−^ foetal liver-derived macrophages (FLDM). TLR stimulation of *Ripk1*^−/−^ FLDM, unlike RIPK3-deficient macrophages, caused low levels of spontaneous caspase-1 and IL-1β activation, which was not further enhanced by Cp.A (Fig. 6b and Supplementary Fig. 4a,b). *Ripk1*^−/−^ FLDMs also displayed a reduced capacity for inflammasome priming (Fig. 6b), and TNF production following LPS stimulation (Fig. 6d and Supplementary Fig. 4c).

We have recently demonstrated that LPS-induced IL-1β activation in the absence of RIPK1 is RIPK3 dependent^34^. Similar to RIPK3-dependent IL-1β activation in caspase-8 or IAP-depleted macrophages, the caspase-1 and IL-1β secretion (but not TNF secretion) observed in LPS-treated *Ripk1*^−/−^ FLDMs was abrogated by glyburide inhibition of NLRP3 (Fig. 6c–e).

*Ripk1*^−/−^ FLDMs were also killed by LPS (or TNF) stimulation (Fig. 6f, Supplementary Fig. 4d–h, and Video 2), which correlated with caspase-1 and caspase-8 processing and activation (Supplementary Fig. 4h). TNF-induced death of *Ripk1*^−/−^ FLDMs was prevented by caspase inhibition (Supplementary Fig. 4d,e). Remarkably, LPS-induced death of *Ripk1*^−/−^ FLDMs, like IL-1β secretion, was blocked in *Ripk1*^−/−^*Ripk3*^−/−^ cells (Fig. 6f, Supplementary Fig. 4g and Video 2). Therefore, in response to LPS, RIPK1 expression is required to limit RIPK3 activation of NLRP3–caspase-1 and cell death.

### TRIF and IAPs regulate RIPK3 and MLKL ubiquitylation

**Figure 7:**
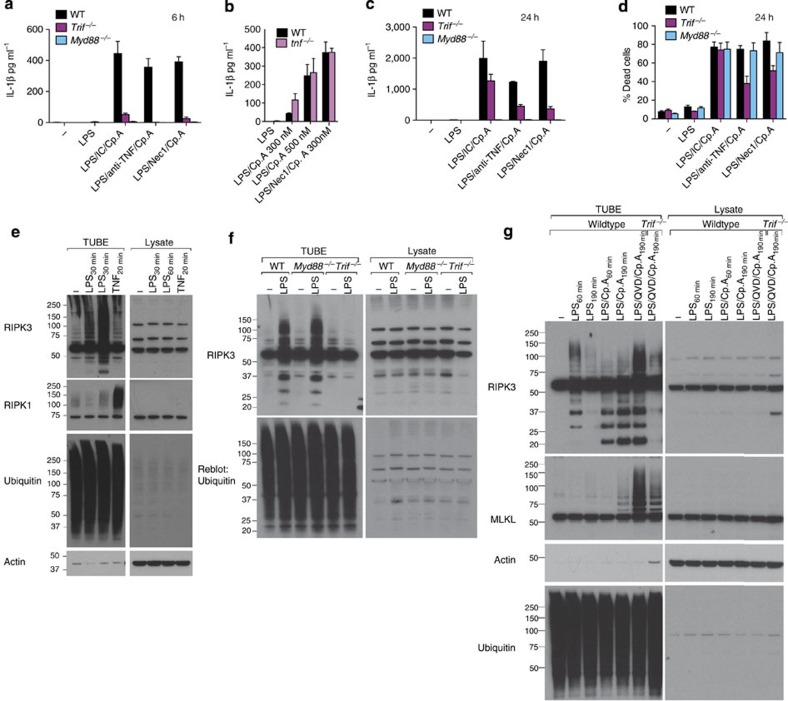
TRIF and IAPs regulate LPS-induced ubiquitylation of RIPK3 and MLKL. (**a**) WT, *Myd88*^−/−^ and *Trif*^−/−^ BMDM were cultured with neutralizing antibodies to TNF (anti-TNF [XT-22] 20 μg ml^−1^), isotype control antibodies (IC [GL113] 20 μg ml^−1^) or Nec-1 (50 μM), and primed for 2 h with LPS, as indicated. Cells were then cultured with Cp.A (500 nM), and IL-1β levels were assayed in supernatants by ELISA at 6 h. *n*=3 mice per group, mean+s.e.m., representative of one of three experiments. (**b**) WT and *Tnf*^−/−^ BMDM were primed for 3 h with LPS, and treated with Nec-1 (50 μM) as indicated. Cells were then cultured with Cp.A (500 nM), and after 6 h IL-1β secretion was assayed by ELISA. *n*=3 mice per genotype, mean+s.e.m., one of three experiments. (**c**,**d**) WT, *Myd88*^−/−^ and *Trif*^−/−^ BMDM were cultured with neutralizing antibodies to TNF (anti-TNF [XT-22] 20 μg ml^−1^), isotype control antibodies (IC [GL113] 20 μg ml^−1^) or Nec-1 (50 μM), and primed for 2 h with LPS, as indicated. Cells were then cultured with Cp.A (500 nM) for 24 h, and (**c**) IL-1β levels assayed in supernatants by ELISA and (**d**) cell death (% Dead cells) assessed by PI and FACS. *n*=3 mice per group, mean+s.e.m., representative of one of three experiments. (**e**) WT BMDM were treated with 50 ng ml^−1^ LPS (30 and 60 min) or 100ng ml^−1^ TNF (20 min) and ubiquitylated proteins were isolated by TUBE and analyzed by immunoblot. One of two experiments. (**f**) WT, *Myd88*^−/−^, and *Trif*^−/−^ BMDM were treated with LPS (100 ng ml^−1^) for 60 min and endogenous ubiquitylated proteins isolated by TUBE and analyzed by immunoblot. One of three experiments. (**g**) WT and *Trif*^−/−^ BMDM were pre-incubated with Q-VD-OPh (20 μM) for 40 min, cultured with Cp.A 500 nM and subsequently treated with LPS (50 ng ml^−1^) for 60 or 190 min as indicated. Endogenous ubiquitylated proteins were isolated by TUBE and analyzed by immunoblot. One of three experiments.

LPS–TLR4 signalling can directly engage RIPK3 by RHIM–RHIM homotypic interactions with the adaptor protein TRIF^28^,^31^. Consistent with this, early LPS- and Cp.A-induced IL-1β secretion required TRIF, and was not blocked by Nec-1 inhibition of RIPK1 or TNF deficiency (Fig. 7a,b). After 24 h of LPS and Cp.A treatment, however, autocrine TNF production contributed to IL-1β secretion and cell death, because TRIF-deficient BMDM displayed increased IL-1β secretion and cell death, which was reduced by the addition of neutralizing TNF antibody or Nec-1 (Fig. 7c,d and Supplementary Fig. 5a–c). On the other hand, Myd88 is essential for LPS-induced NF-κB and inflammasome priming (that is, pro-IL-1β induction) and therefore its deletion abrogated all LPS- and Cp.A-induced IL-1β secretion (Fig. 7a,c and Supplementary Fig. 5a–c), but not LPS and Cp.A killing (Fig. 7d).

The above data suggests that IAPs may regulate a LPS–TLR4–TRIF–RIPK3 complex to limit RIPK3 activation. Considering that purified IAPs can ubiquitylate RIP kinases *in vitro*^35^, we utilized tandem-ubiquitin binding entities (TUBEs) to purify ubiquitylated proteins from macrophages (Fig. 7e–g). TNF stimulation of WT BMDM induced rapid RIPK1 ubiquitylation, as anticipated, but did not markedly ubiquitylate RIPK3 (Fig. 7e). In contrast, LPS stimulation induced significant RIPK3 ubiquitylation within 30–60 min, while it was less efficient at causing RIPK1 ubiquitylation (Fig. 7e). Notably, efficient LPS-induced RIPK3 ubiquitylation observed after 60 min was dependent on TRIF (Fig. 7f) and IAPs (Fig. 7g). Because LPS alone does not induce macrophage death, this suggests that although TRIF directly engages RIPK3 following LPS treatment, IAPs may ubiquitylate RIPK3 to facilitate pro-survival responses.

To investigate if TRIF and IAPs may regulate ubiquitylation of the necrosome (RIPK3–MLKL), we performed TUBEs on BMDM treated with LPS, Q-VD-OPh and Cp.A. Remarkably, after 3 h of necroptotic stimulation, we observed significant ubiquitylation of both RIPK3 and MLKL, which at this time point was largely TRIF-dependent (Fig. 7g and Supplementary Fig. 5d). This implies that IAP proteins suppress E3 ligases that ubiquitylate RIPK3 and MLKL on induction of necroptosis to promote necrosome-induced NLRP3 activity, death and/or regulate necrosome stability.

### IAPs suppress spontaneous inflammatory joint disease

**Figure 8:**
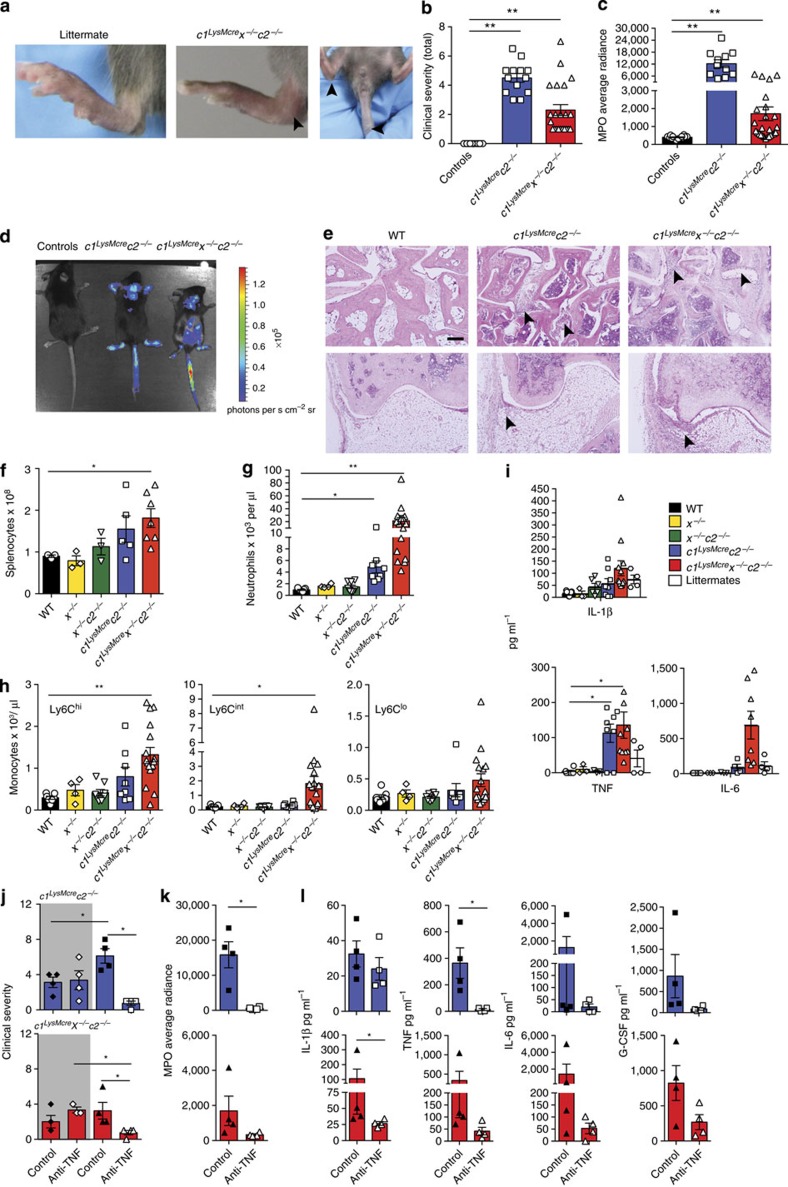
Loss of IAPs in myeloid cells drives TNF-dependent inflammatory joint disease. (**a**–**e**) WT, littermate controls and *c1*^*LysMcre*^*x*^−/−^*c2*^−/−^ and *c1*^*LysMcre*^*c2*^−/−^ mice were monitored for spontaneous inflammatory disease. (**a**) Photos depicting examples of a normal ankle (littermate) and severe swelling in the ankle and tail of a *c1*^*LysMcre*^*x*^−/−^*c2*^−/−^ mouse. (**b**) Clinical severity score of swelling and redness (0–3 for severity per limb). Data are the clinical score of individual mice (out of 12). Mean±s.e.m., **P<0.01, Mann–Whitney two-sample rank test. (**c**) Neutrophil activity assessed by myeloperoxidase (MPO) average radiance of four limbs per mouse, measured using an IVIS spectrum after bioluminescent luminol injection. Data are the mean of individual mice. Mean±s.e.m., **P<0.01, Mann–Whitney two-sample rank test. (**d**) Representative bioluminescent images of MPO activity in control and indicated mutant mice. (**e**) Representative histological examples of disease in WT control, and diseased *c1*^*LysMcre*^*x*^−/−^*c2*^−/−^ and *c1*^*LysMcre*^*c2*^−/−^ ankle (top) and knee (bottom) tissue. Magnification, × 10; scale bar, 100 μM. (**f**) WT and IAP mutant splenocyte counts. Symbols indicate individual mice and data are the mean±s.e.m. *P<0.05, Student’s two-tailed *t*-test. (**g**,**h**) WT and IAP mutant mice peripheral blood (**g**) neutrophil and (**h**) monocyte subset number were analyzed by flow cytometry. Symbols indicate individual mice. Mean±s.e.m., *P<0.05, **P<0.01, Student’s two-tailed *t*-test. (**i**) Levels of cytokines, IL-1β, TNF and IL-6 were measured in the serum of WT and IAP mutant mice by ELISA. Symbols indicate individual mice Data show the mean±s.e.m. *P<0.05, Student’s two-tailed *t*-test. (**j**–**l**) *c1*^*LysMcre*^*x*^−/−^*c2*^−/−^ and *c1*^*LysMcre*^*c2*^−/−^ mice were treated with anti-TNF monoclonal antibody (XT-22) or isotype control for 3 weeks, after which (**j**) clinical severity (grey shading indicates pre-treatment clinical scores) and (**k**) MPO activity in limbs and (**l**) serological cytokine levels were assessed. Symbols represent individual mice, mean±s.e.m. *P<0.05, Mann–Whitney two-sample rank test (**j**,**k**) or Student’s two-tailed *t*-test (**l**).

We next sought to determine if IAPs act together to suppress inflammatory disease and cytokine production *in vivo*, similar to *in vitro*. Because IAP deletion can be embryonic lethal due to excessive RIPK1/RIPK3 signalling^17^, we compared *c1*^*LysMcre*^*x*^−/−^*c2*^−/−^ mice^23^ and *c1*^*LysMcre*^*c2*^−/−^ mice, where cIAP1 is deleted in the myeloid cell compartment only. Unexpectedly, both IAP mutant mice presented with spontaneous inflammatory arthritis (Fig. 8a–e). Joint disease was more severe in the *c1*^*LysMcre*^*c2*^−/−^ mice, as evidenced by clinical scores (Fig. 8b), and measurement of neutrophil activity in limbs (Fig. 8c). *In vivo* myeloperoxidase (MPO) imaging demonstrated that both articular and para-articular tissues were inflamed in *c1*^*LysMcre*^*x*^−/−^*c2*^−/−^ and *c1*^*LysMcre*^*c2*^−/−^ mice, including the spine, paws, knees, tail and mandible, but only the *c1*^*LysMcre*^*x*^−/−^*c2*^−/−^ mice appeared to have dermal inflammation (Fig. 8d). Histological examination confirmed inflammatory cell infiltration and destructive changes in multiple joints from *c1*^*LysMcre*^*x*^−/−^*c2*^−/−^ and *c1*^*LysMcre*^*c2*^−/−^ mice (Fig. 8e and Supplementary Fig. 6a). Other common changes in *c1*^*LysMcre*^*x*^−/−^*c2*^−/−^ and *c1*^*LysMcre*^*c2*^−/−^ mice included splenomegaly and signs of splenic architecture disruption (Fig. 8f and Supplementary Fig. 6a).

Inflammatory joint disease was related to myeloid cell dysfunction in both *c1*^*LysMcre*^*x*^−/−^*c2*^−/−^ and *c1*^*LysMcre*^*c2*^−/−^ mice, as mice reconstituted with BM from *c1*^*LysMcre*^*x*^−/−^*c2*^−/−^ mice presented with weight loss and exhibited mild arthritis. In comparison, *c1*^*LysMcre*^*c2*^−/−^ BM chimeras presented with more severe inflammatory arthritis (Supplementary Fig. 6b–d).

Consistent with severe inflammatory disease in *c1*^*LysMcre*^*c2*^−/−^ and *c1*^*LysMcre*^*x*^−/−^*c2*^−/−^ mice, both harboured increased inflammatory cell numbers, with elevated neutrophil and monocyte numbers, particularly the inflammatory Ly6c^hi^ subset (Fig. 8g,h and Supplementary Fig. 6e). Elevated serum cytokines were also observed in mice lacking all three IAPs in myeloid cells, particularly IL-1β and IL-6, while *c1*^*LysMcre*^*c2*^−/−^ mice had elevated TNF (Fig. 8i and Supplementary Fig. 6f). These observations correlate with our *in vitro* findings, showing that XIAP is important for repressing IL-1β activation induced by LPS or TNF. Cytokine levels were most likely attributable to myeloid cells, as suggested by elevated serum and joint cytokines in IAP knockout BM chimeric mice (Supplementary Fig. 6g,h). Furthermore, WT myeloid cell responses to LPS and IAP inhibition *in vitro* also revealed that IL-1β and TNF were mainly derived from macrophages and inflammatory Ly6c^hi^ monocytes (Supplementary Fig. 7).

### Joint disease in IAP-deficient mice is TNF dependent

TNF is a common pathological factor in human arthritic disease^36^. We therefore examined if the elevated TNF observed in *c1*^*LysMcre*^*c2*^−/−^ and *c1*^*LysMcre*^*x*^−/−^*c2*^−/−^ mice was driving arthritic pathology. Indeed, we found that treatment of ~3-week-old *c1*^*LysMcre*^*c2*^−/−^ *and c1*^*LysMcre*^*x*^−/−^*c2*^−/−^ mice with neutralizing TNF antibody reduced clinical manifestations including, clinical arthritis and neutrophil MPO activity in limbs (Fig. 8j,k). TNF inhibition also promoted increased body weight and growth, and reduced splenomegaly (Supplementary Fig. 6i–k). Examination of serum cytokine profiles following TNF blockade revealed reduced TNF (and granulocyte-colony stimulating factor (G-CSF) in *c1*^*LysMcre*^*c2*^−/−^ mice, but more importantly reduced IL-1β, TNF, IL-6 and G-CSF in *c1*^*LysMcre*^*x*^−/−^*c2*^−/−^ mice (Fig. 8l). Therefore, *in vivo* TNF drives systemic inflammation on cIAP1/2 loss alone, or together with XIAP, but loss of XIAP is required for TNF to drive high levels of IL-1β production *in vivo.*

### RIPK3 but not MLKL promotes arthritis chronicity

*In vitro* IAP suppression can drive RIPK3 and caspase-8-dependent production of IL-1β, and similarly, *in vivo* IAP loss activates RIPK3 refs 17, 23 and causes spontaneous inflammatory arthritis. We therefore evaluated whether IAP deficiency would also exacerbate K/B × N serum transfer arthritis, which is a murine arthritis model that recapitulates many of the innate-immune cell driven features of human rheumatoid arthritis^37^. Pathology is dependent on caspase-1-independent IL-1 production^38^ (Supplementary Fig. 8a), and is partially TNF dependent^39^ (Supplementary Fig. 8b,c).

**Figure 9:**
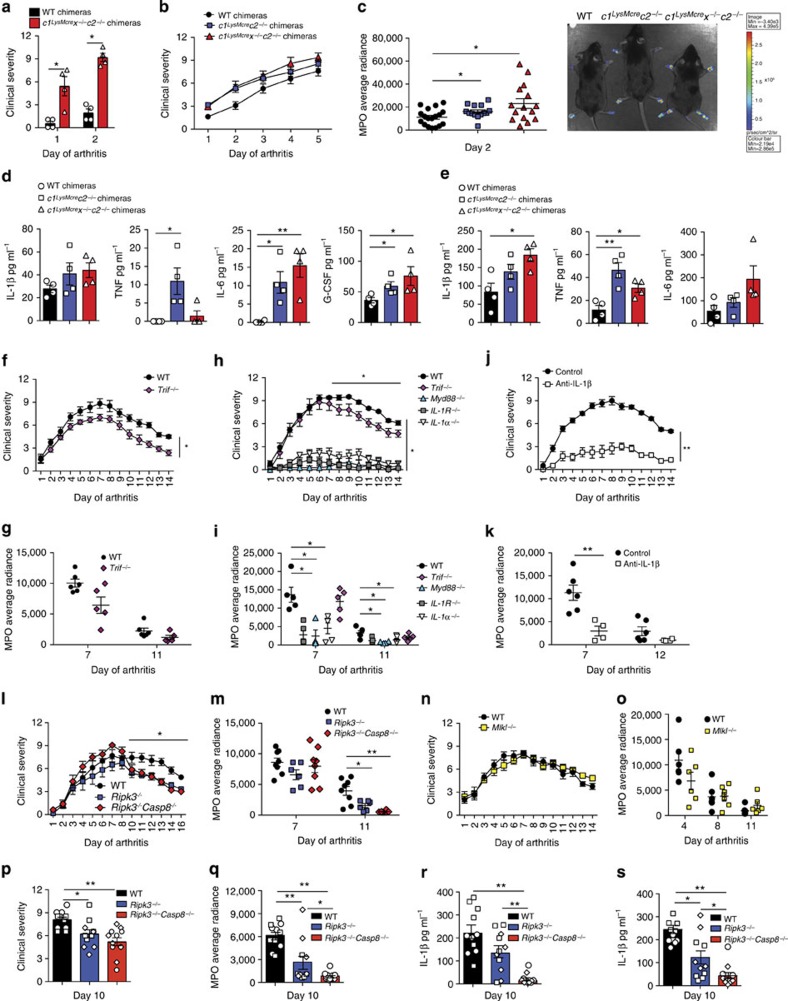
RIPK3 deficiency, but not MLKL loss, decreases innate K/B × N arthritis chronicity. (**a**–**e**) WT, *c1*^*LysMcre*^*c2*^−/−^ and *c1*^*LysMcre*^*x*^−/−^*c2*^−/−^ BM chimeras were injected with 100 μl K/B × N pathogenic serum, and (**a**,**b**) clinical severity of disease (0–3 per limb) was measured; data are represented as mean±s.e.m., (**a**) *n*=4–5 mice per group, *P<0.05, (**b**) *n*≥11 mice per group (WT versus *c1*^*LysMcre*^*x*^−/−^*c2*^−/−^, *P*=0.0196; WT versus *c1*^*LysMcre*^*c2*^−/−^, *P*=0.083). *P* values were calculated using the Mann–Whitney two-sample rank test. (**c**) MPO activity was measured in limbs of mice at day 2 of model. Data show individual mouse MPO average radiance, and a representative bioluminescent image of MPO levels in arthritic mice. Mean±s.e.m., *P<0.05, Mann–Whitney two-sample rank test. (**d**,**e**) Serum (**d**) and ankle joint secretions (**e**) from mice at day 5 of K/B × N arthritis were analyzed for cytokine levels by ELISA. Symbols represent individual mice, mean±s.e.m. *P<0.05, **P<0.01, Student’s two-tailed *t*-test. (**f**–**i**) WT (*n*=5–6/ experiment), *IL-1 R*^−/−^ (*n*=5), *IL-1α*^−/−^ (n =5), *Myd88*^−/−^ (*n*=4) and *Trif*^−/−^ (*n*=5–6 per experiment) mice were injected with 100 μl K/B × N serum and (**f**,**h**) clinical severity and (**g**,**i**) MPO activity (average radiance of individual mice) measured. Mean±s.e.m. *P<0.05, **P<0.01. (WT versus *Trif*^−/−^, total clinical course (**f**) 0.03, (**h**) NS, not significant; resolution phase (**h**) *P*=0.0079, (**f**) 0.0173). *P* values were calculated using the Mann–Whitney two-sample rank test. (**j**,**k**) WT mice were treated with 200 μg antibodies to IL-1β (B122, *n*=4 mice) or control polyclonal hamster antibody (*n*=6 mice) on days −1, 0, 2, 4, 6, 8 and 10 after injection of 100 μl K/B × N pathogenic serum. Mice were evaluated (**j**) daily for clinical severity, and (**k**) MPO activity (average radiance) was measured in limbs on days 7 and 12. Mean±s.e.m. **P<0.01, Mann–Whitney two-sample rank test. One of two experiments. (**l**,**m**) WT, *Ripk3*^−/−^ and *Ripk3*^−/−^*Caspase-8*^−/−^ mice (*n*≥6 mice per group) were injected with 100 μl K/B × N serum. (**l**) Clinical severity, (**m**) MPO activity (average radiance) in limbs of individual mice. Mean±s.e.m. Representative of at least one of two independent experiments. (WT versus *Ripk3*^−/−^, clinical course NS, resolution phase *P*=0.008; WT versus *Ripk3*^−/−^*Caspase-8*^−/−^ clinical course NS, resolution phase *P*=0.0075). *P<0.05, **P<0.01, Mann–Whitney two-sample rank test. (**n**,**o**) WT and *Mlkl*^−/−^ mice (*n*=6 mice per group) were injected with 100 μl K/B × N serum. (**n**) Clinical severity and (**o**) MPO activity in limbs of individual mice are shown. Mean±s.e.m. (**p**–**s**) WT, *Ripk3*^−/−^ and *Ripk3*^−/−^*Caspase-8*^−/−^ mice (*n*=5–6 mice per group) were injected with 100 μl K/B × N serum and during the resolution phase of disease (day 10) analyzed for (**p**) clinical severity, (**q**) MPO activity in limbs, and IL-1β levels measured in (**r**) serum and (**s**) ankle joint secretions. Data symbols are individual mice and different symbols within each group indicate separate experiments. Mean±s.e.m. *P<0.05, **P<0.01. *P* values were calculated using the Mann–Whitney two-sample rank test (**p**,**q**) or the Student’s two-tailed *t*-test (**r**,**s**).

We generated compound IAP mutant BM chimeric mice for arthritis experiments to avoid the severe systemic inflammation in *c1*^*LysMcre*^*c2*^−/−^ and *c1*^*LysMcre*^*x*^−/−^*c2*^−/−^ mice. Deficiency of all three IAPs (*c1*^*LysMcre*^*x*^−/−^*c2*^−/−^ BM chimeras), but not XIAP and cIAP2, in the myeloid compartment led to accelerated and exacerbated K/B × N arthritis (Fig. 9a–c and Supplementary Fig. 8f,g). This was associated with high serum and local cytokine production, particularly IL-1β and TNF (Fig. 9d,e). In comparison, K/B × N serum injection revealed similar levels of arthritis in *c1*^−/−^*, c2*^−/−^, *x*^−/−^ and WT mice (Supplementary Fig. 8d,e).

Considering IAPs regulate RIPK3 activity, and suppress K/B × N arthritis severity, we sought to determine if a TLR–TRIF–RIPK3 axis could regulate K/B × N arthritis via caspase-8 or MLKL signalling. K/B × N arthritis disease chronicity, but not initiation, is dependent on TLR4 ref. 40. Similar to this observation, we also documented reduced K/B × N arthritis persistence, but not initiation, in TRIF-deficient mice (Fig. 9f–i). Importantly, K/B × N arthritis clinical severity and inflammatory profile was dependent on IL-1R expression, the essential IL-1R signalling adaptor Myd88, and both the IL-1R ligands, IL-1α and IL-1β (Fig. 9h–k).

Remarkably, injection of WT, *Ripk3*^−/−^, *Ripk3*^−/−^*Caspase-8*^−/−^ and *Mlkl*^−/−^ mice with K/B × N serum revealed accelerated disease resolution in mice lacking RIPK3 alone, or in combination with caspase-8 (Fig. 9l–q and Supplementary Fig. 8h–k). In contrast, MLKL deficiency failed to alter disease development or resolution (Fig. 9n,o and Supplementary Fig. 8j,k). Associated with accelerated disease resolution in *Ripk3*^−/−^ and *Ripk3*^−/−^*Caspase-8*^−/−^ mice (day 10, Fig. 9p,q) were marked reductions in IL-1β levels in serum, and more importantly, ankle joint secretions (Fig. 9r,s).

Collectively, these data document substantial *in vivo* evidence that the clinical chronicity of K/B × N arthritis is dependent on a TLR–TRIF–RIPK3–IL-1β axis that occurs independent of the RIPK3 substrate and essential necroptotic effector, MLKL.

## Discussion

**Figure 10:**
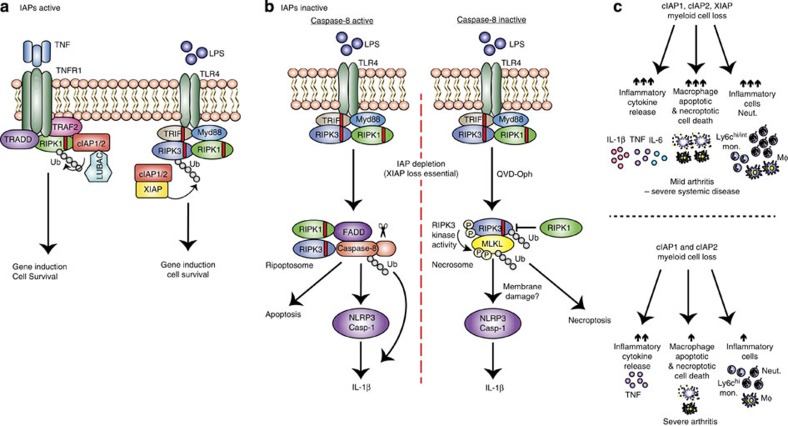
Model for how XIAP and cIAPs repress inflammatory cytokine production, apoptosis and necroptosis. (**a**) When IAPs are present, TNFR1 or TLR–TRIF signalling results in IAP-mediated ubiquitylation of RIPK1 or RIPK3, respectively, to propagate pro-survival signals and gene induction. (**b**) If IAPs are inactivated but caspase-8 is present (left panel), LPS stimulation induces the ripoptosome platform to activate caspase-8 (ubiquitylated). Caspase-8 can (i) trigger apoptosis, (ii) cleave pro-IL-1β directly into its mature form or (iii) promote NLRP3-associated caspase-1 activation, by a mechanism yet to be defined. Alternatively, if both IAPs and caspase-8 are inactive (right panel), LPS induces the formation of the RIPK3–MLKL necrosome that, in addition to causing necroptotic cell death, activates the NLRP3 inflammasome. This necroptotic pathway is associated with RIPK3 and MLKL ubiquitylation, which may control RIPK3/MLKL signalling and/or stability. (**c**) Genetic loss or inhibition of cIAPs alone or XIAP and cIAPs in myeloid cells cause differential effects on cell death, cytokine production and haematopoiesis leading to spontaneous arthritis. Loss of all three IAPs leads to spontaneous systemic inflammatory disease, featuring mild joint inflammation. Disease is associated with increased cytokine release, including IL-1β and TNF, as well as apoptotic and necroptotic cell death and the accumulation of innate inflammatory cells. In contrast, loss of cIAP1/2 causes severe arthritis that is associated specifically with enhanced TNF levels and only modest effects on haematopoiesis.

We report that even in the absence of MLKL, RIPK3 can promote activation of IL-1β both *in vitro* and in a mouse model of inflammatory arthritis, *in vivo.* However, under conditions of chemical or genetic caspase-8 suppression, the necrosome (RIPK3–MLKL) can also engage the NLRP3 inflammasome to activate IL-1β (summarized in Fig. 10). Importantly, both pathways appear to be cell intrinsic, demonstrating that necroptotic cell death and the release of damage-associated molecular patterns (DAMPs) need not be the only drivers of RIPK3-induced inflammation.

RIPK3 and its substrate, MLKL, are essential for TLR and TNF receptor-mediated necroptosis^7^,^8^,^9^,^10^,^11^,^12^,^28^,^31^,^41^, and RIPK3 has been implicated in the inflammatory response in different disease models, including viral infection, retinal degeneration, brain and renal injury, and atherosclerosis^6^. However, in these and other disease models the reliance on MLKL for pathology and inflammatory cytokine production has yet to be evaluated^6^. Recently it was reported that RIPK3 is required for optimal transcription of LPS-induced cytokines in dendritic cells^42^ and MLKL-independent NLRP3 inflammasome activation in response to dsRNA viral infection^43^. Similarly, we have shown that RIPK3 can drive TNF production independent of MLKL in response to IAP antagonist treatment^23^. Therefore, considering these reports and our findings here, it will be important to assess which RIPK3-driven inflammatory diseases can occur independently of MLKL and necroptosis, particularly with the emergence of potential therapeutics targeting RIPK3 kinase activity and MLKL itself^31^,^44^,^45^.

The loss of IAPs triggers the spontaneous formation of a RIP kinase, FADD, caspase-8, cFLIP signalling complex termed the ripoptosome^20^,^21^. In response to TNF or TLR ligation this complex can signal apoptosis, or in the absence of caspase-8 RIPK3–MLKL-mediated necroptosis, or as we have demonstrated, IL-1β activation^22^. IAPs harbour RING domains that can act as E3 ubiquitin ligases, of which, cIAP1/2 ubiquitylate RIPK1 in response to TNF stimulation to prevent RIPK1 signalling cell death, and to propagate pro-survival signals^3^,^35^,^46^. Together with a recent study^47^, our data describe a more intricate picture, because in macrophages XIAP appears to be a key repressor of TNF- and LPS-induced caspase-8 activation, necroptosis and caspase-1/IL-1β activity. Although our data, and that of others^48^, clearly demonstrate that cIAP1/2 co-operate with XIAP to repress these ripoptosome-driven responses, it is notable that the RING domain of XIAP is important^47^. In this regard, it will be interesting to determine the requisite substrates of XIAP RING E3 ligase activity.

In IAP-depleted cells, LPS engagement can activate RIPK3 signalling, with at least three distinct observable outcomes. First, in macrophages RIPK3 can promote caspase-8 activity and apoptosis independent of its kinase function and substrate MLKL. Unlike some cell lines where cIAP1/2 loss is sufficient for rapid TNF killing, the co-deletion of XIAP in macrophages is obligate for efficient TNF- or LPS-induced caspase-8 activity and death. Notably, TNF stimulation and IAP antagonism or RIPK3 over-expression, can also signal RIPK3–caspase-8-mediated apoptosis^49^,^50^. More recently, it was reported that chemicals targeting the RIPK3 kinase domain^45^, or expression of D161N kinase dead RIPK3 *in vivo*^51^, induces spontaneous apoptosis via RIPK3 RHIM domain-mediated recruitment and activation of a ripoptosome complex. Interestingly, kinase dead RIPK3 *per se* does not drive caspase-8 activation, and the generation of viable RIPK3 kinase dead mice that are unable to signal necroptosis but retain apoptotic potential^45^, will help decipher what role RIPK3-induced apoptosis and RIPK3–caspase-8 activation of NLRP3 may play *in vivo*.

Second, active caspase-8, resulting from IAP loss and LPS-induced RIPK3 signalling, promotes NLRP3-associated caspase-1 activation and IL-1β maturation. This function is distinct from the ability of caspase-8 to directly cleave pro-IL-1β into its mature bioactive form^22^,^52^. In RIPK3-deficient macrophages, caspase-8 is required for the LPS and Cp.A-induced NLRP3–caspase-1 activation that accumulates over time, since this was completely abrogated by caspase-8 inhibition or deletion. Although the mechanism by which caspase-8 can activate NLRP3-associated caspase-1 is unclear, it has recently been proposed that caspase-8 may influence caspase-1 proteolysis following NLRP3 engagement^33^,^53^,^54^. Significantly, however, caspase-8 engagement of NLRP3–caspase-1 appears to be stimulus-specific, because we only observed this phenomenon in IAP-depleted cells, and not cells treated with the canonical NLRP3 activator nigericin.

Third, if caspase-8 function is reduced LPS stimulation of IAP-depleted cells triggers RIPK3 kinase activity and activation of MLKL. Other than necroptosis, under these conditions we observed that MLKL is essential for NLRP3–caspase-1 activation. This finding is consistent with the observation that following caspase-8 deletion in dendritic cells, small interfering RNA depletion of MLKL reduced RIPK3-driven NLRP3 activity^25^. Recent studies report that MLKL can disrupt plasma membrane integrity to modulate ion influx, such as Ca^2+^ ref. 14, which is also one means proposed to trigger NLRP3 activation^55^,^56^,^57^,^58^, and is therefore a candidate mechanism by which MLKL signalling causes inflammasome activation.

Auto-inflammatory syndromes, such as neonatal onset multi-organ inflammatory disease that features arthropathy have been linked with mutations in the NACHT domain of NLRP3, while inflammatory arthropathies, such as rheumatoid arthritis and ankylosing spondylitis, have been associated with mutations in the TNF superfamily^24^,^59^,^60^. We now find that mice with myeloid-specific loss of all three IAPs or only cIAP1/2 exhibit spontaneous inflammatory cytokine secretion and joint disease. As expected, based on numerous murine and human studies implicating TNF in arthritis^36^, we found that TNF was the master regulator of cytokine production and inflammatory arthritis in IAP-deficient mice. Why arthritis was worsened by cIAP1/2 co-deletion, compared with the triple IAP-deficient mice, remains unclear. However, loss of all IAPs causes severe systemic inflammatory features reminiscent of endotoxic shock, or mice expressing constitutively active NLRP3 ref. 61, thus the accumulation of myeloid cells in the periphery may divert the innate-immune response. Alternatively, excessive macrophage cell death within joint tissues upon loss of all IAPs could limit inflammatory cell influx, akin to depleting joint macrophages in arthritis models^62^.

In rheumatoid arthritis, disease chronicity and worsened prognosis is associated with joint macrophage accumulation^63^. In mice and humans, IL-1 is a major pathogenic cytokine in arthritis, including the innate cell-mediated, caspase-1-independent, K/B × N serum transfer arthritis model^38^,^64^. Neutrophil proteases have been linked with IL-1β activation during disease initiation^64^, however, the pathways driving monocyte/macrophage secretion of IL-1β responsible for arthritis chronicity, remain ill-defined. Importantly, our findings reveal that a TLR–TRIF–RIPK3–caspase-8 signalling pathway may promote K/B × N arthritis disease persistence by driving IL-1β production via transcriptional induction, and/or cleavage induced activation. Although dependent on RIPK3, this mechanism occurs independent of necroptosis, since MLKL deficiency did not alter arthritis pathogenesis. Enhanced K/B × N arthritis disease resolution observed upon TRIF or RIPK3 deletion phenocopies TLR4 mutant mice^40^, suggesting that host danger molecules present in arthritic joints may promote rheumatic flares via TLRs present on synovial macrophages^65^. Therefore, for optimal therapeutic targeting of RIPK3 in inflammatory diseases, such as arthritis, it will be important to carefully evaluate the bifurcation of RIPK3-induced necroptosis and DAMP-driven inflammation versus cell intrinsic-induced cytokine production and activation.

## Methods

### Mice

Mice were housed under standard conditions at WEHI. All procedures were approved by the WEHI Animal Ethics Committee. Female and male mice were 3–12 weeks old at the time of experimentation, with the exception of K/B × N arthritis experiments that were performed on 6–8-week-old male mice (BM reconstituted male mice were used at 12–14 weeks). All animal numbers used for each experiment are reported in the figure legends. The inducible K/B × N arthritis model and spontaneous inflammation in the IAP deficient mice have 90–100% penetrance. 4–12 mice were used per genotype per experiment, sufficient to calculate statistical significance. Mouse strains and sources are detailed in the Supplementary Methods.

### IAP antagonist compounds

The IAP antagonist compounds and their antagonism of cIAPs versus XIAP were previously described^26^. The compound nomenclature has been changed in the current study, with compounds 2, 1, 9, 10 and 8 described in ref. 26 now designated as Cp.A, 711 (birinapant), 030, 455 and 851, respectively. Compounds 031 and 883 are reported for the first time here. Their synthesis and structure are described in detail in the Supplementary Methods.

### Cell culture

Bone marrow cells were harvested from femoral and tibial bones, or foetal liver cells sieved from E13.5 livers. To generate macrophages, BM and foetal liver cells were cultured in bacterial Petri dishes for 6 days in Dulbecco’s modified Eagle’s medium (DMEM) containing 8% foetal bovine serum, 50 U ml^−1^ penicillin and 50 μg ml^−1^ streptomycin (complete media) and supplemented with 20% L929 conditioned media (37 °C, 10% CO_2_). For isolation of neutrophils and monocyte populations, cells were stained with fluorochrome-conjugated anti-mouse Ig antibodies—CD11b, F4/80, Ly6G and Ly6C—and sorted using a Moflo instrument.

BMDM and foetal liver-derived macrophages were plated in tissue culture plates overnight at 1–2 × 10^5^ per well (96 wells) or 3–5 × 10^5^ per well (24 wells). Neutrophils, Ly6c^hi^, Ly6c^int^ and Ly6c^lo^ monocytes were plated at 1–2 × 10^5^ per well (96 wells). Cells were primed for 3 h with ultra-pure LPS (20 ng ml^−1^, Invivogen), Fc-human TNF (100 ng ml^−1^, in-house) or Pam_3_Csk_4_ (P_3_Cys; 2–2.5 μg ml^−1^, Invivogen), and stimulated as specified with Smac-mimetics (500 nM, unless specified), Q-VD-OPh (10–20 μM, R&D Systems), Nec-1 (50 μM, in-house), GSK872 (ref. 31; RIPK3 inhibitor, 1 μM, in-house), Glyburide (100–200 μM, Sigma), Alum (300 μg ml^−1^, ThermoScientific), Nigericin (5–10 μM, Sigma) and ATP (5 mM, Sigma). Supernatants were routinely collected at 5–6 h (45 min for ATP and Nigericin) and 24 h post stimulation for analysis of cytokines and lactate dehydrogenase release (Promega). In some cases, cells were harvested from tissue culture/non-tissue culture treated 24-well plates using 5 mM EDTA/phosphate-buffered saline (PBS) to assess viability (propidium iodide (PI) uptake) by flow cytometry (see below). A viable cell gate was generated to determine the percentage of dead cells. Alternatively cells were lysed in reducing sample buffer for analysis by immunoblot.

### Time lapse imaging

BMDMs and FLDMs were plated at 0.75–1 × 10^5^ per well in L929 conditioned media in a 96-well optical-bottom plate (Nunc) and allowed to adhere overnight at 37 °C, 10% C0_2_. Media were removed and cells were labelled with CellTracker Green CMFDA (CTG, 1 μM, Life Technologies, ThermoScientific) in serum-free DMEM for 30 min at 37 °C, 10% CO_2_. Following two to three washes in phenol-red-free DMEM, cells were incubated in 8% FCS phenol-red-free DMEM. Cells were then stimulated with LPS (20 ng ml^−1^), as indicated, and treated in the final 20 min of priming period with Q-VD-Oph (10–20 μM). PI (1 μg ml^−1^) and Cp.A (500 nM) were added prior to imaging on a Zeiss Wide scope microscope (37 °C, 10% CO_2_). Cells were imaged (× 10 magnification) from 2–4 h post-LPS stimulation every 30 min, and images were analyzed for number of CTG+ and PI+ cells over time using MetaMorph software image analysis. Movies were generated using Image J software (4 frames per s).

### Cytokine ELISA

IL-1β and IL-18 (R&D Systems, Ebioscience), and TNF and IL-6 (Ebioscience, R&D Systems) ELISA kits and paired G-CSF antibodies and standard (R&D Systems, Peprotech) were used to perform ELISAs on serum, joint fluid and supernatants according to the manufacturer’s instructions.

### Leucocyte counts

Peripheral blood cells from retro-orbital bleeds or cardiac bleeds were counted on an Advia 120 cell analyzer (Bayer Diagnostics, Tarrytown, NY, USA). Spleen and BM preparations were subjected to red blood cell lysis prior to automated (Countess) or manual cell counts using Trypan blue for dead cell exclusion.

### Flow cytometry

Single cell suspensions of blood and BM were prepared. Cells were incubated with Fc block (FcγRIIb/III, 2.4G2) and stained with the following fluorochrome-conjugated anti-mouse Ig antibodies according to the manufacturer’s instructions (BD Biosciences (San Diego, CA, USA), eBioscience (San Diego, CA, USA), Biolegend (San Diego, CA, USA) or the WEHI Monoclonal Antibody facility): CD45.1 (A20), CD45.2 (104), CD11b (Mac-1), F4/80 (A3–1), Ly6G (1A8), GR1 (Ly6C/Ly6G), RB6.8C5 (WEHI), Ly6c (HK1.4), Annexin V and PI. Stained cells were profiled on a LSR1, LSRII or LSR Fortessa instrument (all Becton Dickinson) using CellQuest Software version 3.3 or FACSDiva Software (BD Immunocytometry Systems, North Ryde, Australia) and data were analyzed using in-house developed WEASEL version 2.7 software (WEHI).

### BM reconstitutions

BM was harvested from the femur, tibial and pelvic bones of WT and conditional IAP mutant mice and red blood cells were lysed. C57BL/6 Ly5.1 mice were lethally irradiated (2 × 550R) 3 h apart. Mice received 6–10 × 10^6^ BM cells by i.v. tail injection and were allowed to reconstitute for 3–8 weeks to achieve ~85–90% reconstitution.

### Gross scoring of clinical parameters

WT and IAP compound mutant mice were graded for inflammatory arthritis from 0 (normal) to 3 (severe) for joint inflammation (limbs). For TNF neutralization experiments, mice were scored at 3 weeks of age and matched for disease severity then assigned to receive 10 mg kg^−1^ of anti-TNF monoclonal antibody (XT-22; WEHI Monoclonal Antibody Facility) or isotype control (GL113) three times a week for 3 weeks. Gross parameters, including arthritis (clinical/MPO levels IVIS imaging), weights, body length, spleen weight and serum cytokines, were measured.

### *In vivo* bioluminescent imaging of MPO activity

Mice were injected i.p. with luminol (200 mg kg^−1^) and anaesthetized (isoflurane inhalation) prior to bioluminescence imaging on an IVIS spectrum instrument (Caliper; exposure time 180 s; binning 4, Field of view 12.5 cm) on specified days, based on previous studies^66^. Regions of interest (ROI) were manually selected over front and rear paws using Living Image Software, and identical ROIs were used for time course analysis. For spontaneous disease, mice were imaged front and back. C57BL/6 naïve mice injected with luminol were used to control for background luminescence (~400 average radiance).

### K/B × N serum transfer arthritis

K/B × N arthritis was induced as described^66^. Briefly, 100–200 μl of pooled serum (based on batch testing) from arthritic K/B × N mice was injected i.p. Mice were examined daily for clinical signs of arthritis and paws graded from 0 to 3 for severity of paw/ankle inflammation. In some experiments, mice were imaged for MPO activity in limbs (see above). Weight loss of ~15% for more than 2 consecutive days and/or severe disease was the endpoint for studies (that is, Fig. 9a–c). In some cases, serum was harvested, and ankles were cultured in 0.1% BSA in DMEM for 1–2 h, and supernatants and serum were analyzed by ELISA.

For anti-IL-1β therapeutic studies, mice were treated i.p. 1 day prior to K/B × N arthritis induction with 200 μg anti-IL-1β (B122, Bio X Cell,) or control (polyclonal Armenian hamster IgG, Bio X Cell) antibodies. Mice were then injected (d0) with K/B × N serum and received 200 μg doses of anti-IL-1β or control on days 2, 4, 6, 8 and 10 of the model.

### Histology

Tissues were harvested (for example, spleen, knees, limbs, and tail), fixed in 10% (w/v) neutral buffered formalin, decalcified and embedded in paraffin. Frontal tissue sections were stained with haematoxylin and eosin and assessed by blinded investigators.

### Immunoblotting

Cell lysates and supernatants (reduced and denatured) were separated on 4–12% gradient gels (Invitrogen) and protein transferred nitrocellulose membrane (Amersham) for detection. Membranes were blocked with 5% skimmed milk in PBS containing 0.1% Tween 20 (PBST) for 1–2 h and were then probed overnight with primary antibodies (all diluted 1/1,000 unless noted otherwise) to mouse β-actin (Sigma; A-1978), cIAP1 (1/500 dilution, ALX-803-335;Alexis Bio-chemicals), RIPK3 (Axxora; PSC-2283-c100), RIPK1 (BD Transduction Laboratories; 610458), MLKL (in-house; 3H1), pro and mature IL-1β (R&D Systems; AF-401-NA), pro- and cleaved-caspase-1 (Santa Cruz; sc-514), Adipogen (AG-20B-0042-C100), pro-caspase-8 (in-house), cleaved caspase-8 Asp387 (9429; Cell Signaling) and Ubiquitin (3933; Cell Signaling). Relevant horseradish peroxidase-conjugated secondary antibodies were applied for 1–2 h. Membranes were washed four to six times in PBST between antibody incubations and all antibodies were diluted in PBST containing 5% skimmed milk. Membranes were developed using ECL (Millipore). For images cropped for presentation, the full-size images are presented in Supplementary Figs 9–14.

### TUBE purification

Following specified stimulations, ice-cold PBS-washed BMDMs (10–12 × 10^6^) were harvested in 1,000 μl of lysis buffer (30 mM Tris-HCl (pH 7.4), 120 mM NaCl, 2 mM EDTA, 2 mM KCl, 1% Triton X-100, Roche complete protease-inhibitor cocktail, 1 mM NEM) and lysed on ice for 30 min. Lysates were cleared by centrifugation (14,000*g*, 10 min) and endogenous ubiquitylated proteins were isolated from the soluble lysate at 4 °C for 3–20 h using agarose TUBEs (TUBE1, Life sensors, performed according to the manufacturer’s instructions). Following 4 × washes in lysis buffer, bound proteins were eluted using reducing and denaturing western blot sample buffer.

### Statistical analyses

The Mann–Whitney two-sample rank test was used to analyze the level of significance between mean clinical score, MPO measurements and clinical readouts. The Student’s two-tailed *t*-test (assuming equal variance) was used to compare cytokine levels and for fluorescence activated cell sorting (FACS) analysis. For each test, *P* values <0.05 were considered statistically significant.

## Author contributions

K.E.L. and J.E.V. conceived the project and designed the research; K.E.L., J.E.V., N.K., A.M., M.G., B.A.C., A.A.D., C.H., S.K.S., H.A. and M.R. performed the experiments; S.M.C. designed and synthesized the IAP antagonist compounds; C.A.B. and Y.M. assisted with the interpretation of the IAP antagonist induction of IL-1β data. M.G., B.A.C., W.W-L.W., J.S. and D.L.V. contributed to the interpretation of the results and drafting of the manuscript; W.S.A. provided *Mlkl*^−/−^ mice and S.L.M. provided *Nlrp3*^−/−^ and *caspase-1*^−/−^ mice and commented on the manuscript; I.P.W. provided essential reagents and expertise in arthritis models and commented on the manuscript. K.E.L. and J.E.V. analyzed the data, prepared figures and wrote the manuscript.

## Additional information

**How to cite this article**: Lawlor, K. E. *et al*. RIPK3 promotes cell death and NLRP3 inflammasome activation in the absence of MLKL. *Nat. Commun.* 6:6282 doi: 10.1038/ncomms7282 (2014).

## Supplementary Material

Supplementary Figures, Methods and References Supplementary Figures 1-14, Supplementary Methods and Supplementary References

Supplementary Movie 1 LPS and Cp. A induced cell death. CTG labeled WT, Ripk3-/- and Ripk3-/-Caspase-8-/- BMDM were primed for 2 hrs with LPS (20 ng/ml), and in the final 20 min Q-VD-OPh (20 μM) was added as indicated. Macrophages were then stimulated with Cp.A (500 nM) where indicated and PI added prior to time lapse imaging. Images were taken from 4 hrs post priming at 30 min intervals (green = CTG, red = PI; 4 frames/sec, 10X magnification) and movies made using Image J software.

Supplementary Movie 2 RIPK1 represses RIPK3 induced cell death upon TLR ligation. CTG labelled Ripk1+/+, Ripk1-/-, Ripk3-/- and Ripk1-/-Ripk3-/- FLDM were primed for 1 hr with LPS (20 ng/ml), and in the final 20 min Q-VD-OPh (20 μM) was added as indicated. Macrophages were then stimulated with Cp.A (500 nM) where appropriate and PI added prior to time lapse imaging. Images were taken at 30 min intervals (green = CTG, red = PI; 4 frames/sec, 10X magnification), and movies made using Image J software.
