# Supplementary material for: RIPK3 promotes cell death and NLRP3 inflammasome activation in the absence of MLKL
Source: Nat Commun. 2015 Feb 18;6:6282. doi: 10.1038/ncomms7282 (PMC4346630; doi:10.1038/ncomms7282)
Supplement: Supplementary Figures, Methods and References — Supplementary Figures 1-14, Supplementary Methods and Supplementary References [file ncomms7282-s1.pdf]

Supplementary Information

Supplementary Figures

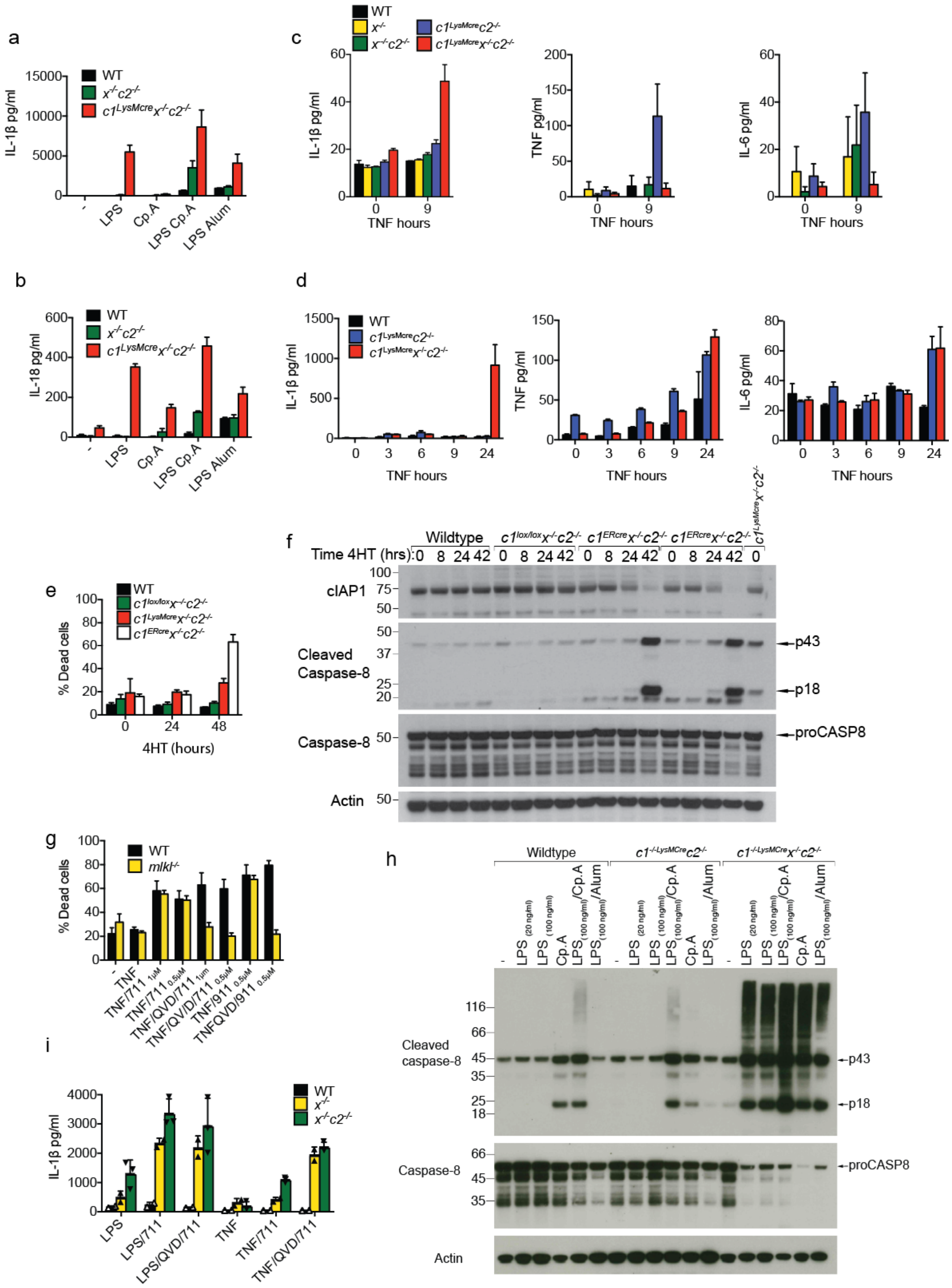

Supplementary Figure 1. IAPs regulate cytokine production and cell death.
(a,b) WT, $x^{-/-}c2^{-/-}$, and $c1^{LysMcre}x^{-/-}c2^{-/-}$ BMDM were primed with LPS for 3 hrs, and stimulated with Cp.A (500 nM) or Alum (300 µg/ml) for a further 6 hrs. (a) IL-1β and (b) IL-18 activation was assayed in supernatants by ELISA. n = 3 mice/group, Mean + SEM. **(c,d)** WT, $x^{-/-}$, $x^{-/-}c2^{-/-}$, $c1^{LysMcre}c2^{-/-}$, and $c1^{LysMcre}x^{-/-}c2^{-/-}$ BMDM were cultured with TNF (100 ng/ml) and over time cell supernatants harvested for analysis of cytokines by ELISA. n = 3 mice/group, mean + SEM.

(e) WT, $c1^{lox/lox}x^{-/-}c2^{-/-}$, $c1^{LysMcre}x^{-/-}$ $c2^{-/-}$, and $c1^{ERcre}x^{-/-}c2^{-/-}$ BMDM were pulsed with 4HT (1000nM) for 16 or 30 hrs and analysed 8 or 12 hrs later, respectively for PI uptake. Data show % dead cells, n = 3 mice all except $c1^{LysMcre}x^{-/-}$ $c2^{-/-}$ (n=2), mean + SD. **(f)** WT, $c1^{lox/lox}x^{-/-}c2^{-/-}$, $c1^{LysMcre}x^{-/-}$ $c2^{-/-}$, and $c1^{ERcre}x^{-/-}c2^{-/-}$ BMDM were cultured with 4HT (1000 nM) and at indicated times lysates analysed by immuno-blot for cIAP1 deletion and caspase-8 processing. **(g)** WT and *Mlkl*$^{-/-}$ immortalised MDFs were cultured with TNF (100 ng/ml), 0.5 or 1 µM 711 and QVD-Oph (10 µM), as indicated, for 24 hrs. Cell death was measured by PI uptake (% dead cells). n = 3 mice, mean ± SEM. Representative of 1 of 3 experiments. **(h)** WT, $c1^{LysMcre}c2^{-/-}$, and $c1^{LysMcre}x^{-/-}c2^{-/-}$ BMDM were primed with LPS, as indicated, and cultured with Cp.A (500 nM) or Alum (300 µg/ml) for 6 hrs and lysates analysed by immuno-blot. **(i)** WT, $x^{-/-}$, $x^{-/-}c2^{-/-}$ BMDM were primed with LPS or TNF for 3 hrs, cultured as indicated with Q-VD-OPh in the final 20 min of priming, and then treated with cIAP1/2 selective inhibitor, 711 (500 nM). IL-1β secretion was measured after 24 hrs by ELISA. Symbols represent individual mice, mean ± SD. Representative of 1 of 3 experiments.

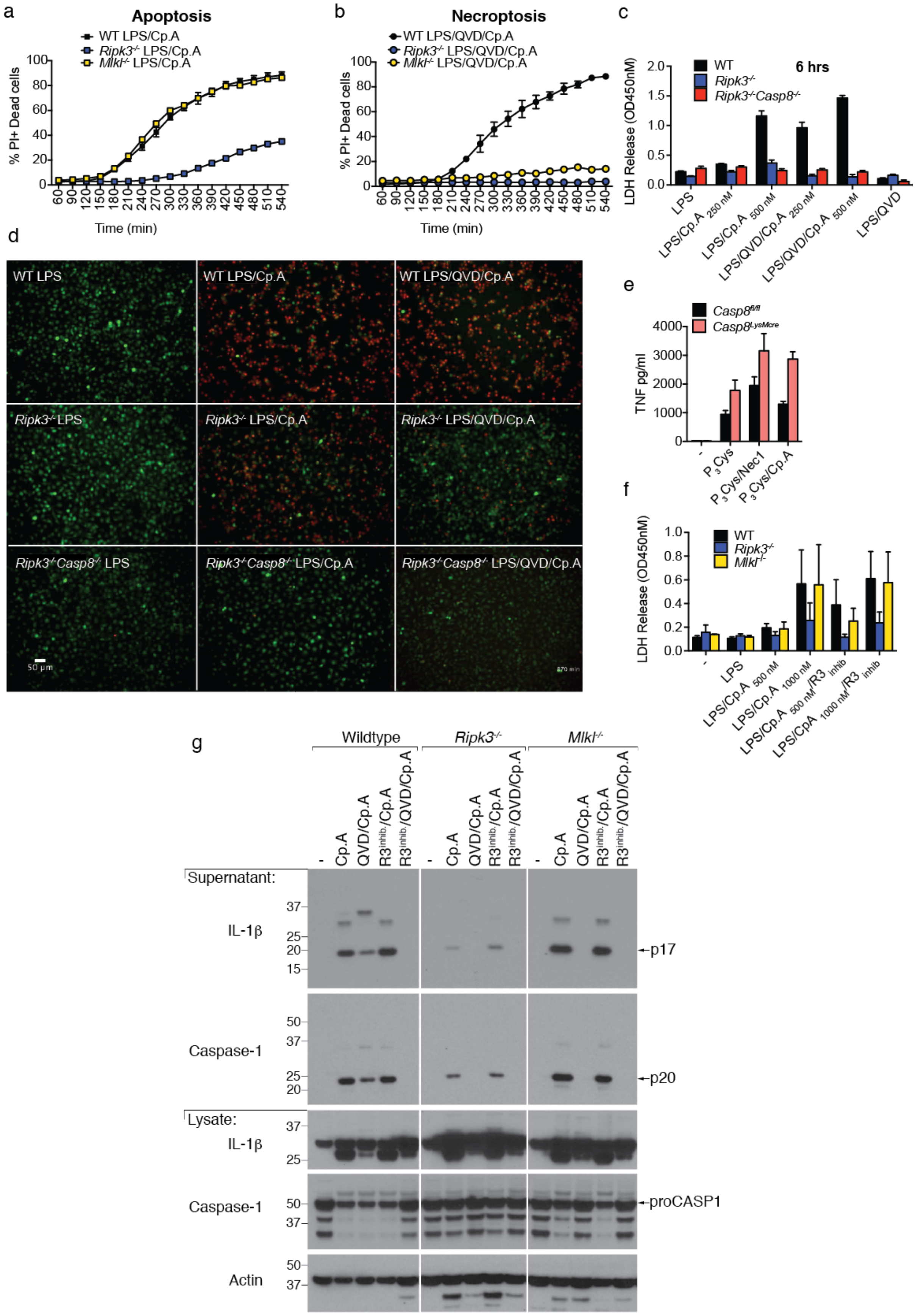

Supplementary Figure 2. RIPK3 induced death and activation of IL-1β.

(a,b) WT, *Ripk3*$^{-/-}$ and *Mlkl*$^{-/-}$ BMDM were labeled with CTG, primed for 3 hrs with LPS and treated with Q-VD-OPh (10 μM) as indicated for the final 20 min of priming. Cells were then stimulated with Cp.A (500 nM) and cell death was measured by PI uptake and time lapse imaging. % PI positive cells, n = 2-3 mice/genotype, Mean ± SD, 1 of 2 experiments. **(c)** WT, *Ripk3*$^{-/-}$, and *Ripk3*$^{-/-}$*Caspase-8*$^{-/-}$ BMDM were primed with LPS (20 ng/ml) for 3 hrs, treated

with Q-VD-OPh in the final 20 min of priming, and Cp.A added, as specified, for 6 hrs. Cell death was measured by LDH release. n = 3 mice, mean + SEM. **(d)** WT, *Ripk3*$^{-/-}$, and *Ripk3*$^{-/-}$*Caspase-8*$^{-/-}$ BMDM were labeled with CTG and primed with LPS for 3 hrs. Q-VD-OPh (20 μM) was added as indicated in the final 20 min of priming prior to Cp.A (500 nM) stimulation and addition of PI. BMDM were imaged from 4 hrs post LPS every 30 min on a Zeiss Live cell observer. Representative images of CTG labeled macrophages (green) and PI+ cells (red) after 14 hrs imaging, n = 3 WT, 4 *Ripk3*$^{-/-}$, and *Ripk3*$^{-/-}$*Caspase-8*$^{-/-}$ mice. 1 of 2 experiments. (See **Video1**). **(e)** WT *caspase-8*$^{lox/lox}$ and *caspase-8*^{LysMcre} BMDM were primed with Pam_3Cys (2.5 μg/ml) for 3 hrs, and Nec1 (50 μM) added as indicated in the final 20 min. Cp.A (500 nM) was then added, where specified, and cells cultured for a further 24 hrs. TNF levels were measured in supernatants by ELISA. n = 3 mice/group, mean + SEM, representative of 1 of 2 experiments. **(f)** WT, *Ripk3*$^{-/-}$ and *Mlkl*$^{-/-}$ BMDM were primed with LPS for 3 hrs, and treated in the final 20 min of priming 1 μM R3 inhib as indicated, prior to addition of Cp.A (500 nM) where shown. Cell viability was measured at 6 hrs by LDH release, n = 3 mice/genotype, mean + SEM. **(g)** WT, *Ripk3*$^{-/-}$ and *Mlkl*$^{-/-}$ BMDM were primed with Pam_3Cys (2.5 μg/ml) for 3 hrs, and treated in the final 20 min of priming with Q-VD-OPh (20 μM) and 1 μM RIP3 kinase inhibitor (R3 inhib) as indicated, prior to addition of Cp.A (500 nM) for 20 hrs. Cell supernatants and lysates were analysed by immunoblot for caspase-1 and IL-1β activation.

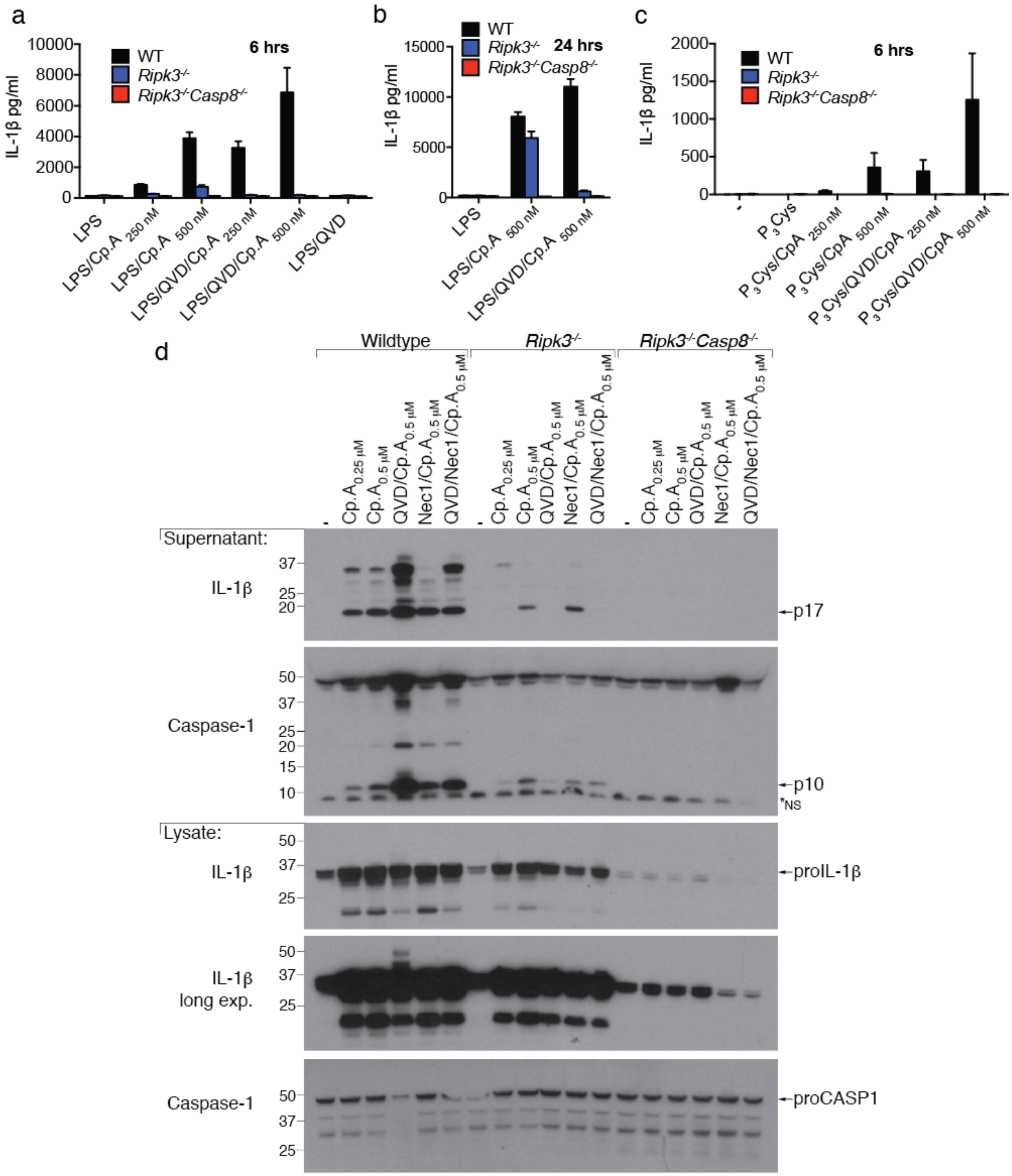

Supplementary Figure 3. IL-1β activation requires RIPK3 and caspase-8.
(a-c) WT, *Ripk3*$^{-/-}$, and *Ripk3*$^{-/-}$*Caspase-8*$^{-/-}$ BMDM were primed with LPS (20 ng/ml) or Pam$_3$Cys (2.5 μg/ml) for 3 hrs, treated with Q-VD-OPh in the final 20 min of priming, and Cp.A added, as specified, for 6 or 24 hrs. IL-1β was measured in supernatants by ELISA. n = 3 mice, mean + SEM. **(d)** WT, *Ripk3*$^{-/-}$, and *Ripk3*$^{-/-}$*Caspase-8*$^{-/-}$ BMDM were primed with Pam$_3$Cys (2.5 μg/ml) for 3 hrs and treated for 20 min with Q-VD-OPh (20 μM), and Nec-1 (50 μM), as indicated prior to Cp.A (500 nM) stimulation for 17 hrs. Supernatants and lysates were analysed by immuno-blot.

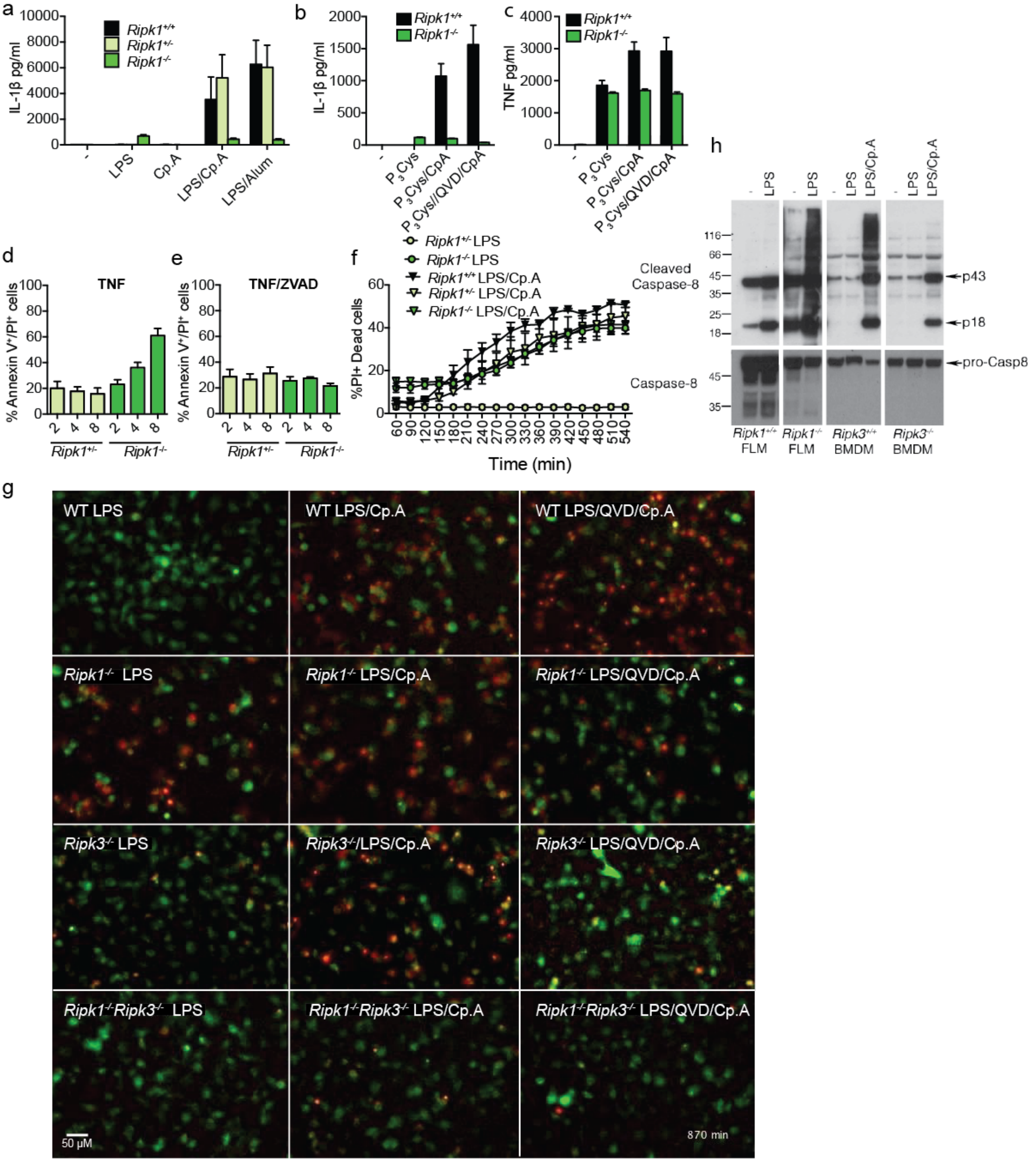

Supplementary Figure 4. RIPK1 represses RIPK3 killing and IL-1β activation.

(a-c) FLDM were primed with LPS (20 ng/ml) or Pam$_3$Cys (2 μg/ml) for 3 hrs, and treated with Q-VD-OPh (20 μM), after which Cp.A (500 nM) or Alum (300 μg/ml) were added for 6 hrs. **(a,b)** IL-1β and **(c)** TNF secretion was measured by ELISA (n=3 mice/genotype). **(d,e)** FLDM were cultured with **(d)** TNF or **(e)** TNF/ZVAD-fmk (50 μM) for 24 hrs and cell death measured by annexin V and PI staining. n = 3 mice/genotype, mean + SD. **(f)** CTG labeled FLDM were primed for 2 hrs with LPS and treated with Cp.A (500 nM). Time lapse imaging was performed every 30 min, and the percentage of PI$^+$ dead cells analysed using MetaMorph. n = 2-3 mice/per genotype, mean ± SD. **(g)** Expanded image (additional LPS/QVD/Cp.A treatment) shown in Fig. 6F. CTG (green) FLDM were primed for 1 hr with LPS (20 ng/ml), and in the final 20 min Q-VD-OPh (20 μM) added. Cells were stimulated with Cp.A (500 nM), and PI (red) added. Representative images of levels of cell death after 14 hrs of imaging, (Magnification 10x; see **Video2**). **(h)** Lysates from BMDM and FLDM after priming with LPS (3 hrs) and then stimulated with Cp.A (500 nM, 6 hrs) were analysed by immuno-blot.

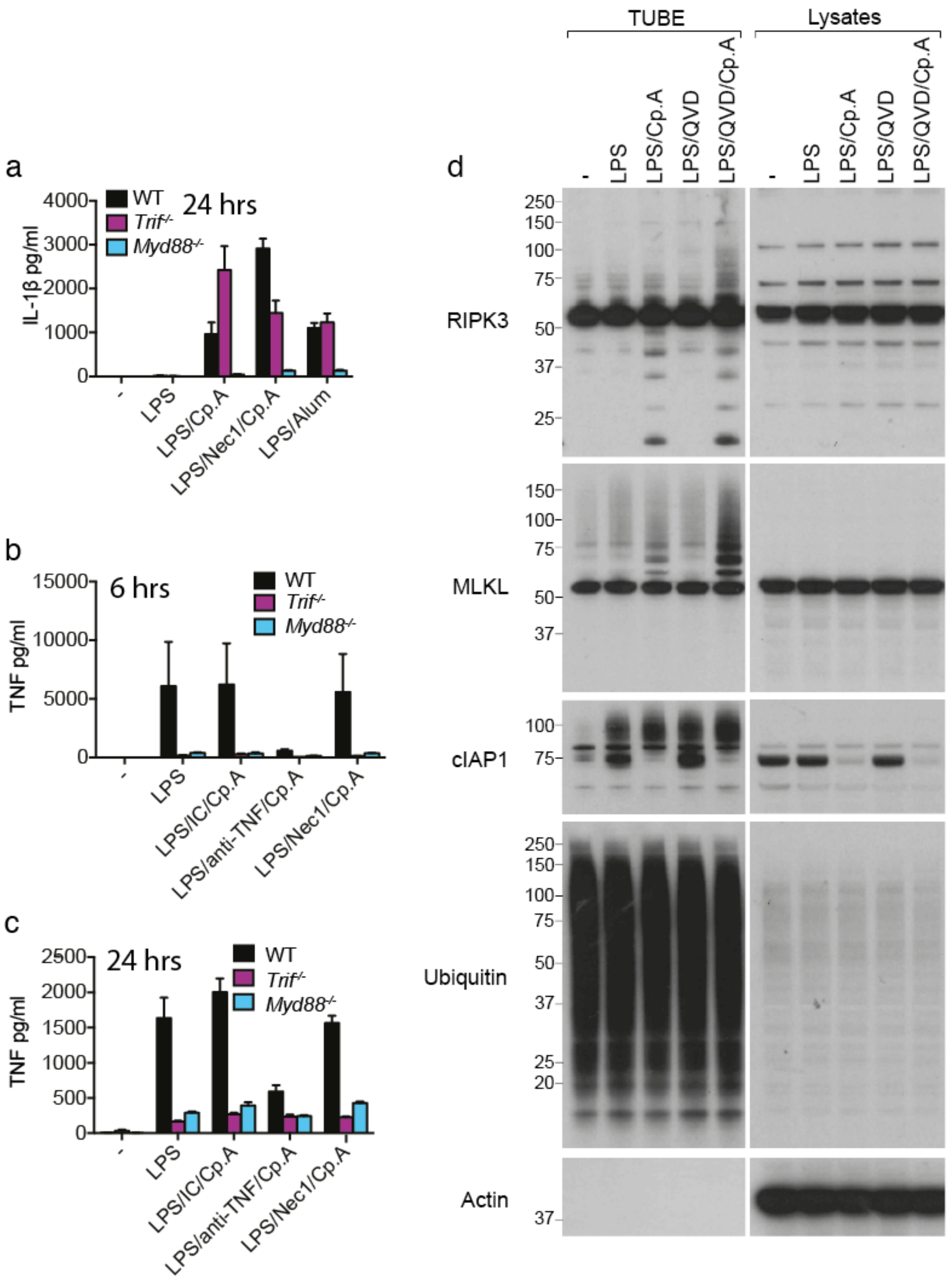

Supplementary Figure 5. Autocrine TNF contributes to Cp.A induced IL-1β secretion. **(a-c)** WT, *Trif*$^{-/-}$ and *Myd88*$^{-/-}$ BMDM were primed with LPS for 2-3 hrs with or without Nec1 (50 μM), anti-TNF (20 μg/ml) or isotype control (IC; 20 μg/ml), prior to stimulation with Cp.A (500 nM) or Alum (300 μg/ml) as indicated. At the designated time points supernatants were analysed for **(a)** IL-1β and **(b,c)** TNF levels by ELISA. n = 3 mice/genotype, mean + SEM, representative of 2-3 experiments. **(d)** WT BMDMs were stimulated with LPS (50 ng/ml), Cp.A (1 μM) and QVD (20 μM) for 3 hrs and 40 min prior to TUBE purification of ubiquitylated proteins and analysis by immuno-blot.

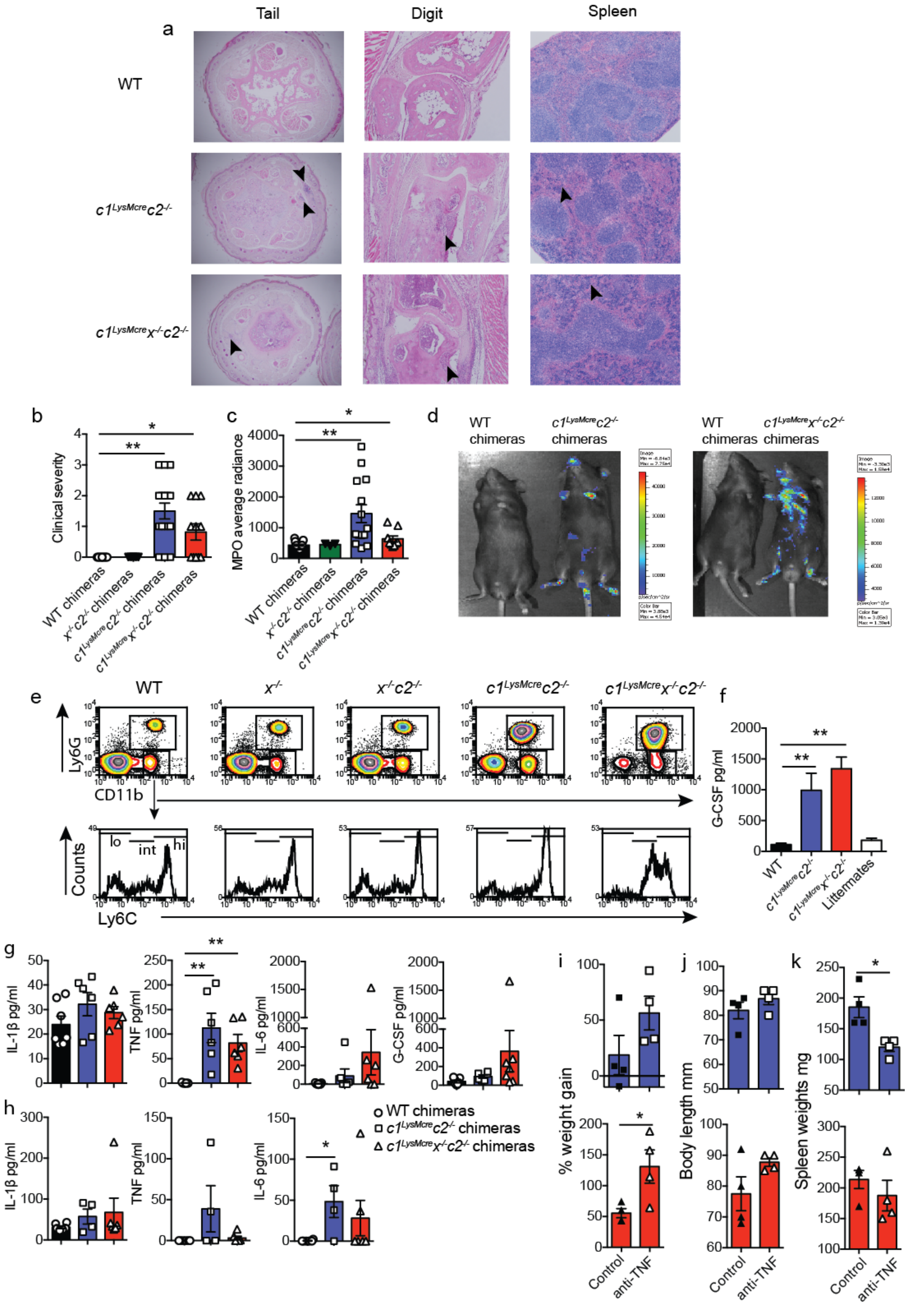

Supplementary Figure 6. Inflammation caused by IAP loss is limited by TNF blockade. **(a)** Representative H&E stained tail (4x Mag), digit (10x Mag) and spleen (10x Mag) of indicated mutants. **(b-d)** Bone marrow was transferred from WT or IAP mutant mice (Ly5.2 background) into lethally irradiated Ly5.1 recipients. Mice were monitored for **(b)** Clinical

joint disease and **(c,d)** MPO measurements in limbs from 3 weeks of age, and **(d)** representative image of MPO activity in bone marrow chimeras. Data show mean of individual mice, mean ± SEM, * P < 0.05, ** P < 0.01, Mann-Whitney two sample rank test. **(e)** Representative flow cytometric contour plots of myeloid populations in the peripheral blood of WT and IAP mutant mice. Neutrophils CD11b^{+}Ly6G^{hi}, inflammatory monocytes CD11b^{+}Ly6G^{-}Ly6C^{hi} and resident monocytes CD11b^{+}Ly6G^{-}Ly6C^{lo}. **(f)** Blood was collected from WT and IAP mutant mice and serum levels of G-CSF measured by ELISA. Mean + SEM, ** P < 0.01, Student's two-tailed *t*-test. **(g)** Serum and **(h)** ankle joint secretions from WT and IAP mutant chimeras were analysed for cytokine levels by ELISA. Symbols are individual mice. Mean ± SEM. * P < 0.05, ** P < 0.01, Student's two-tailed *t*-test. **(i-k)** Cohorts of $c1^{LysMcre}x^{-/-}c2^{-/-}$ and $c1^{LysMcre}c2^{-/-}$ mice treated with anti-TNF (XT-22) or isotype control antibody (GL113) were analysed for clinical improvement including **(i)** % weight gain, **(j)** body length and **(k)** spleen weights after 3 weeks of therapy. Symbols are individual mice, mean ± SEM, * P < 0.05, Mann-Whitney two sample rank test.

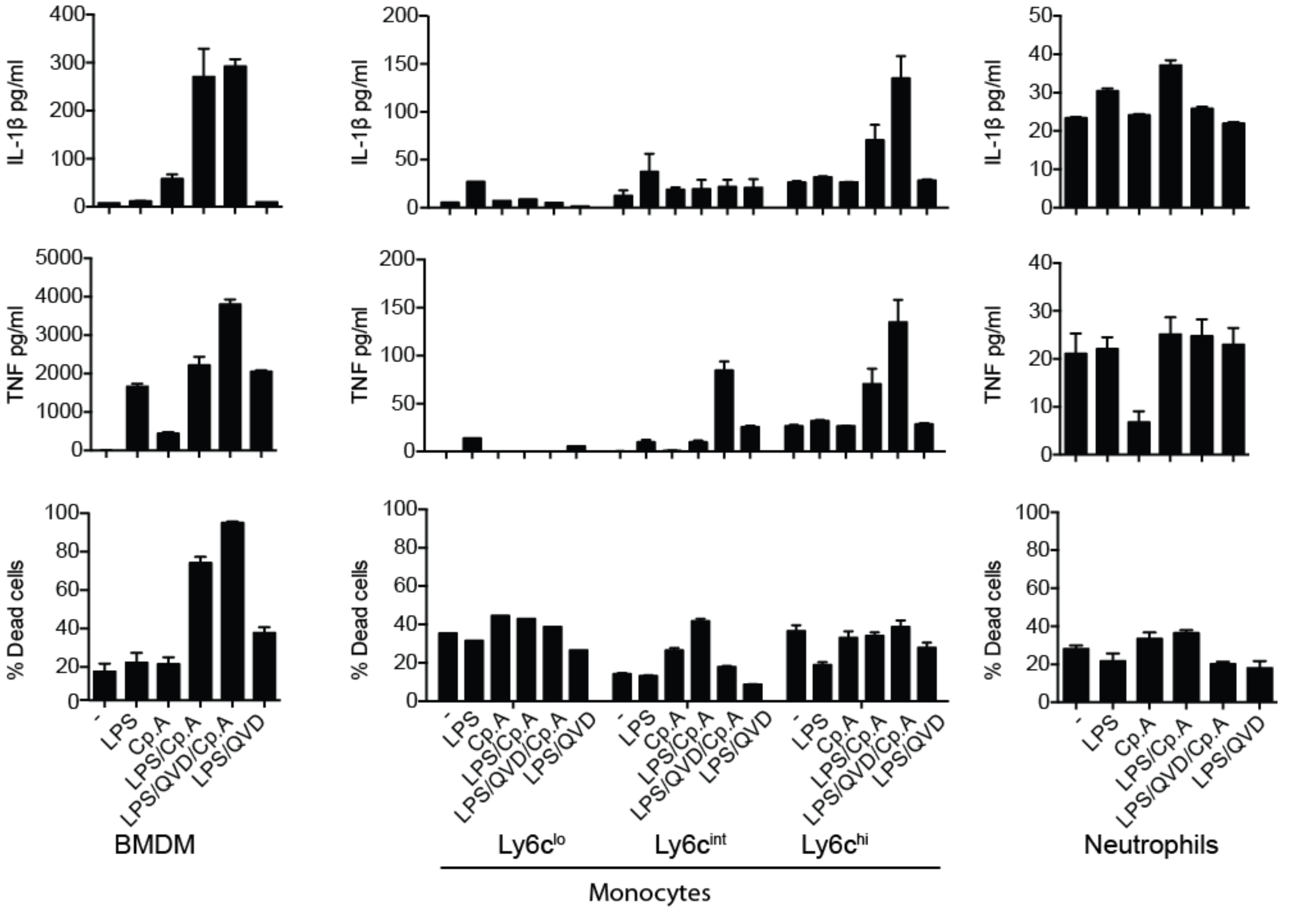

Supplementary Figure 7. Cell types that secrete IL-1β following Cp.A treatment.
BMDM, as well as Ly6c^{hi}, Ly6c^{int}, Ly6c^{lo} monocytes and neutrophils isolated from bone marrow were pre-incubated with LPS (20 ng/ml) as indicated for 3 hrs, then as specified cultured with Q-VD-OPh (20 μM) in the last 20 min of priming and Cp.A (500 nM). After 24 hrs IL-1β and TNF levels were measured by ELISA, and cell death analysed by PI uptake. Data are representative of 3 separate experiments, n = 3 mice/group, with the exception of the Ly6c^{lo} subset where 3 mice were pooled in each experiment. Mean + SEM.

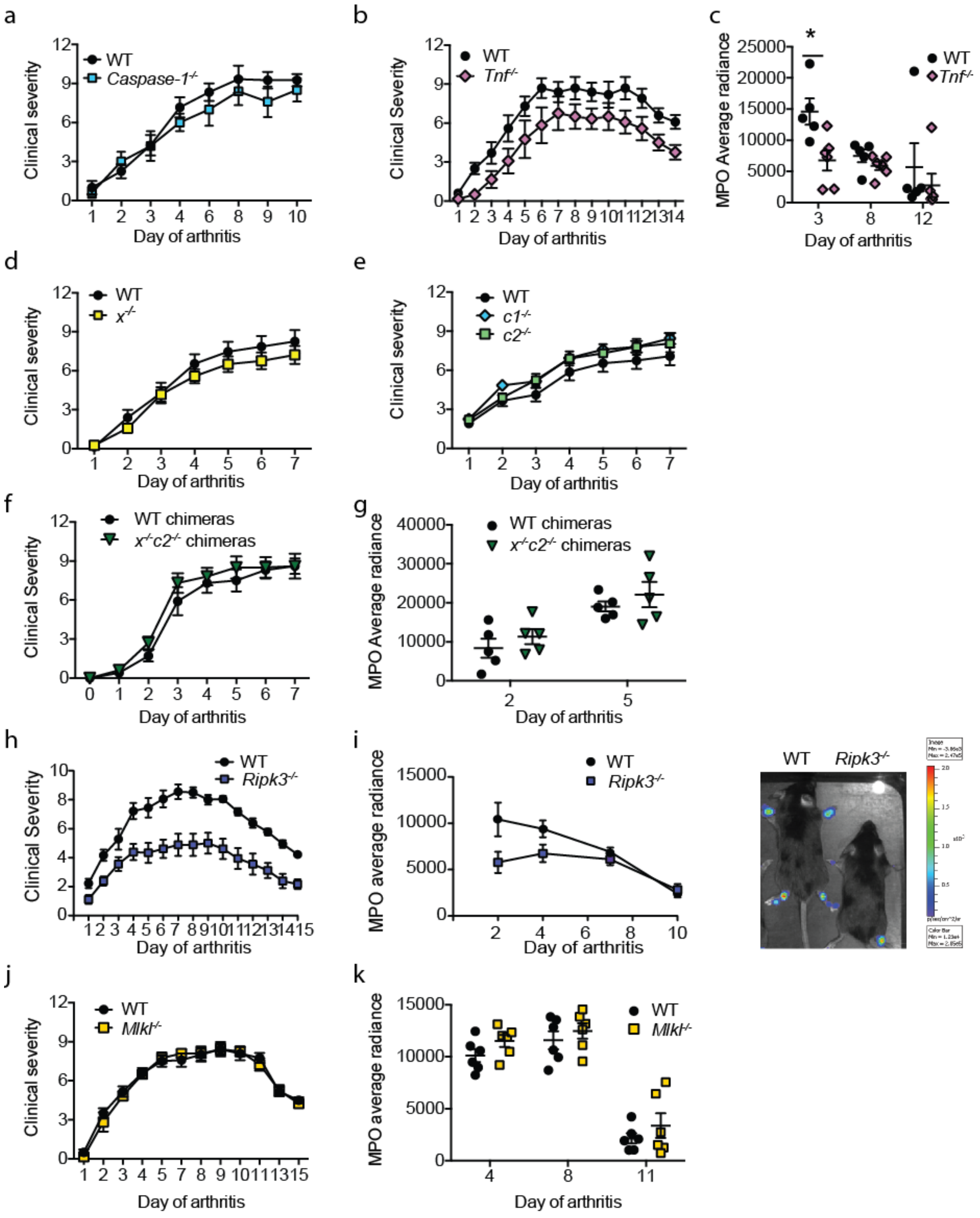

Supplementary Figure 8. K/BxN serum induced arthritis in single IAP deficient mice. **(a-c)** WT, *caspase-1*$^{-/-}$ and *Tnf*$^{-/-}$ mice were injected i.p. with 100 µl K/BxN serum and **(a,b)** monitored for clinical severity (0-3 per limb). **(c)** *In vivo* imaging of MPO activity (average radiance per mouse) in limbs was performed on WT and *Tnf*$^{-/-}$ mice. n ≥ 5-6 mice/group. Mean ± SEM. * P < 0.05, Mann-Whitney two sample rank test. **(d,e)** IAP deficient mice were injected i.p. with 200 µl K/BxN serum and disease severity scored daily. n ≥ 5-6 mice/group, Mean ± SEM. **(f,g)** WT and *x*$^{-/-}$*c2*$^{-/-}$ BM chimeras (n = 5 mice/group) were injected i.p. with 100 µl of K/BxN serum and **(g)** clinical disease severity and **(g)** MPO activity (average radiance per mouse) in limbs of individual mice monitored over time. Mean ± SEM. **(h,i)** WT and *Ripk3*$^{-/-}$mice were injected i.p. with 100 µl of K/BxN serum and **(h)** clinical severity (P = 0.005) and **(i)** MPO activity (average radiance) (P = 0.016) monitored over time. n = 9 mice/group, mean ± SEM. P-values calculated using the Mann-Whitney two sample rank test. **(j,k)** WT and *Mlkl*$^{-/-}$ mice were injected i.p. with 100 µl of K/BxN serum and **(h)** clinical severity and **(i)** MPO activity (average radiance) monitored over time. n = 6 mice/group, mean ± SEM.

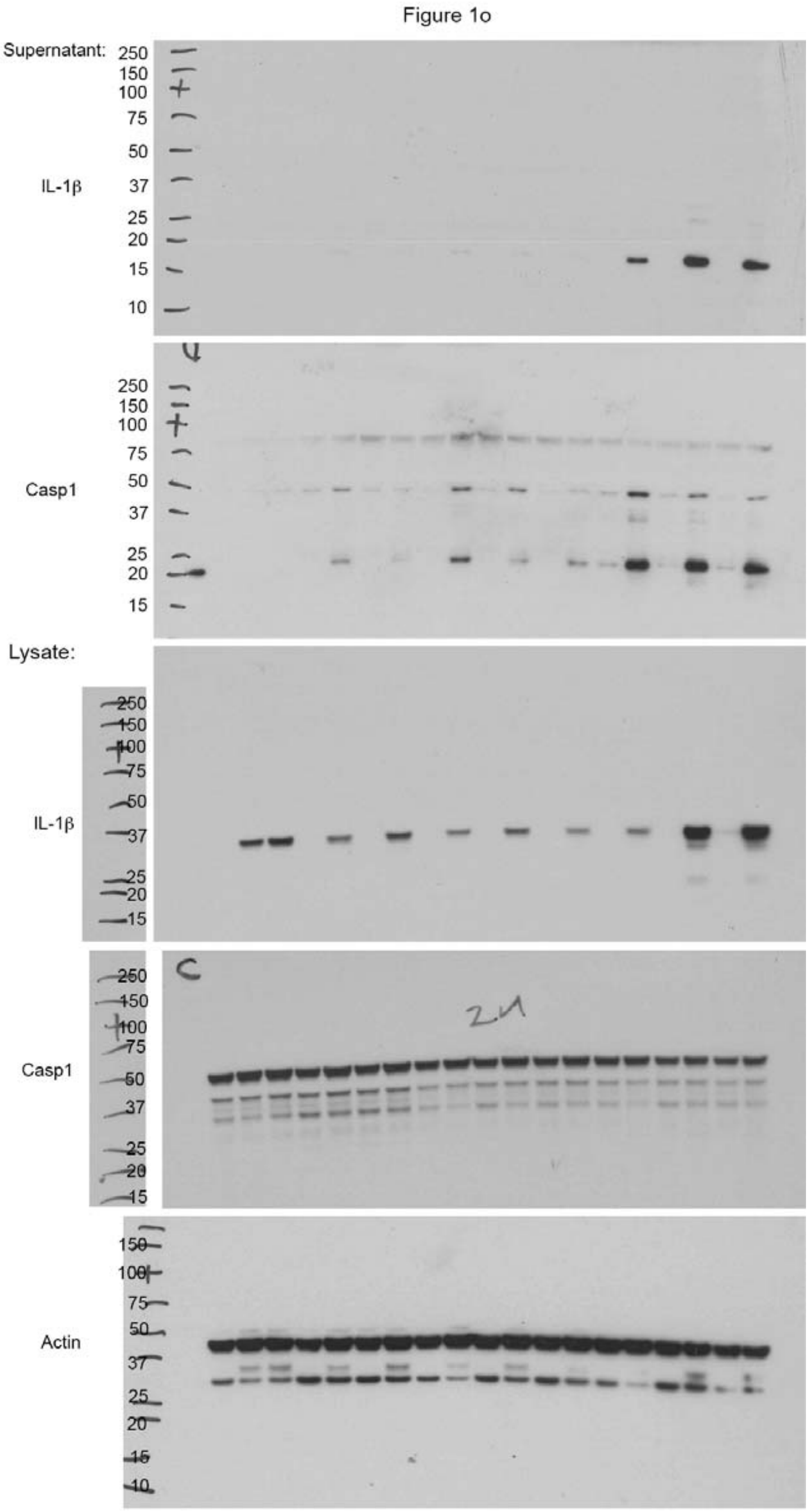

Supplementary Figure 9.
Whole gel scans of immunoblots presented in Figure 1o.

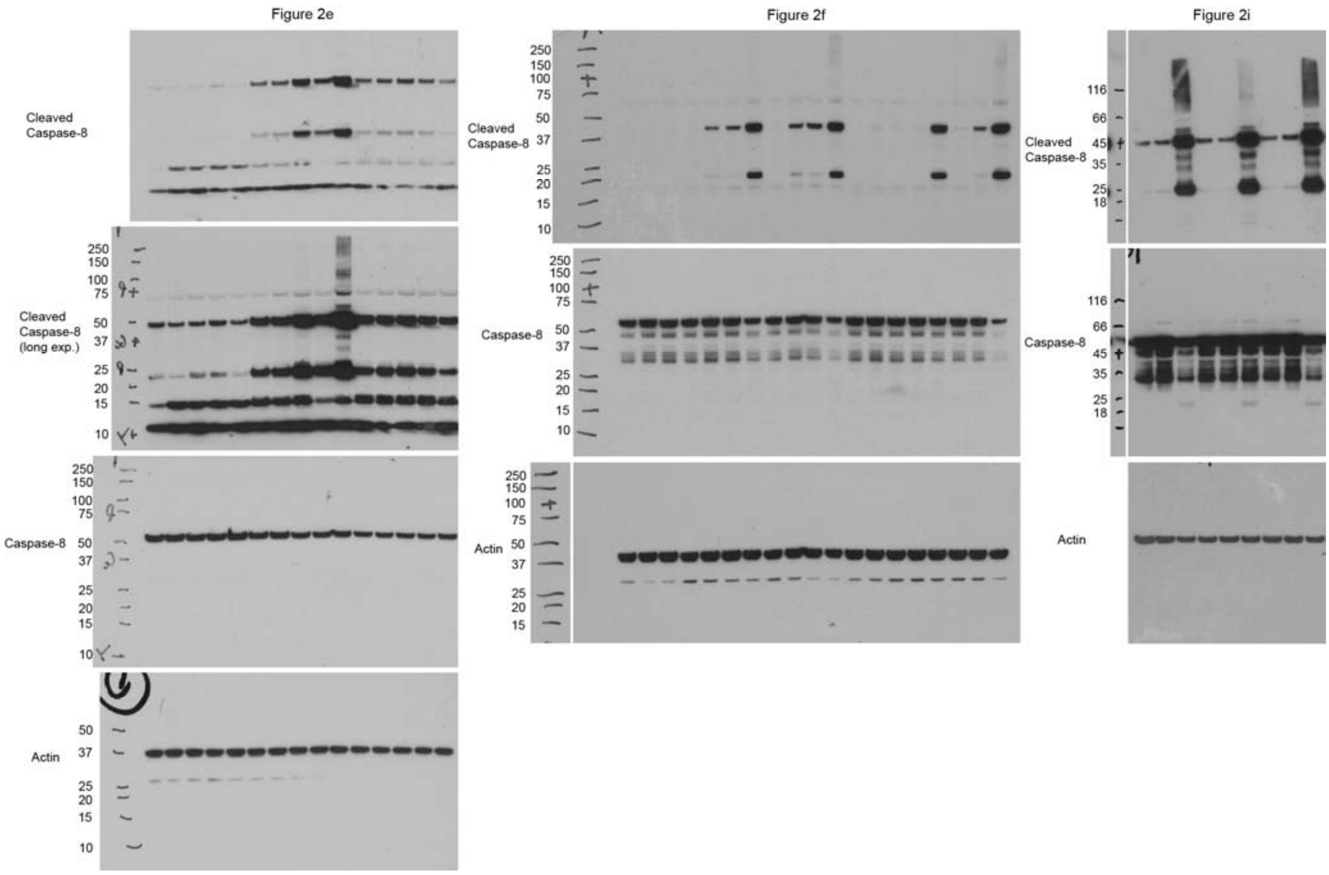

Supplementary Figure 10.
Whole gel scans of immunoblots presented in Figure 2e, 2f and 2i.

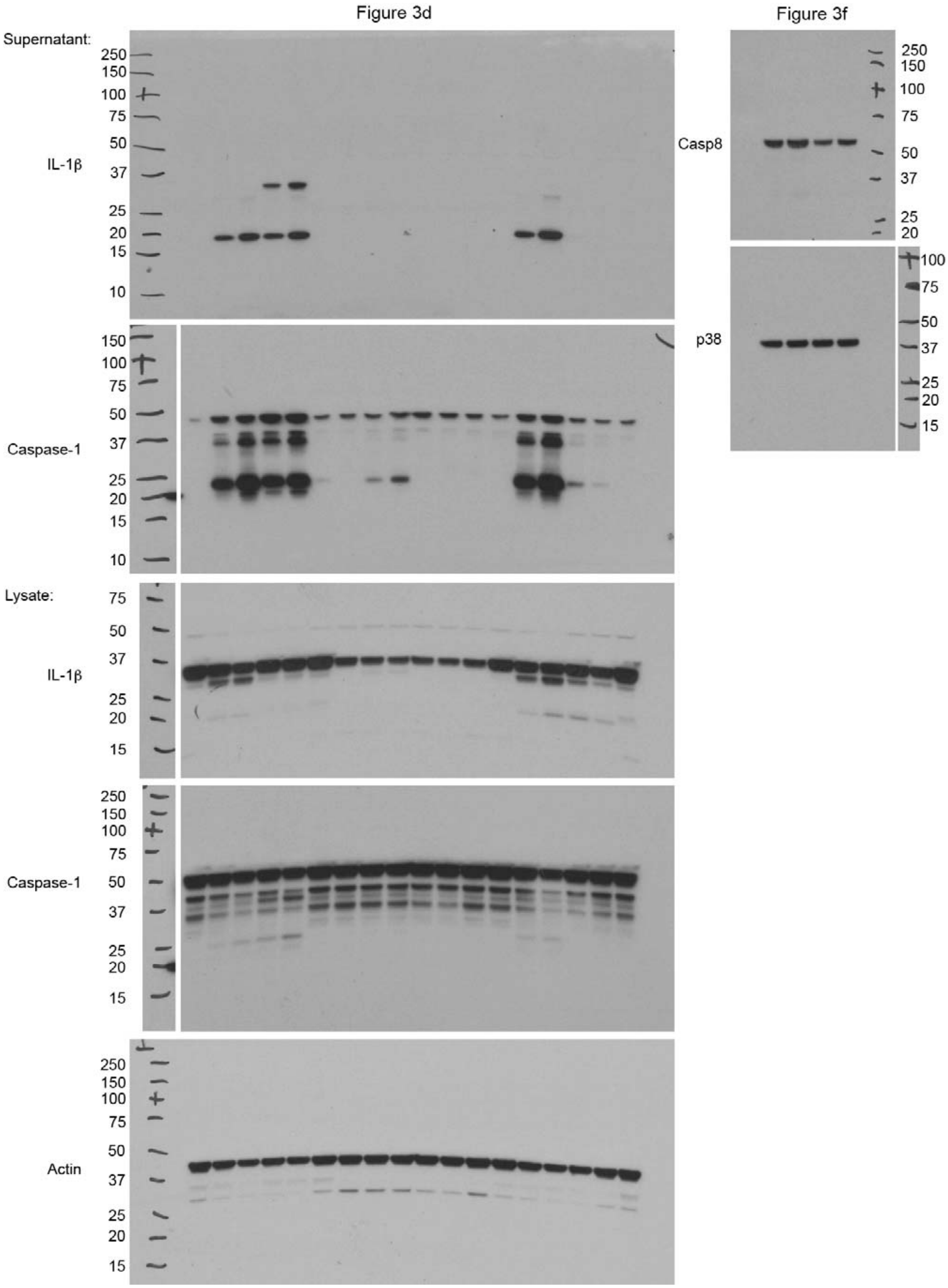

Supplementary Figure 11.
Whole gel scans of immunoblots presented in Figure 3d and 3f.

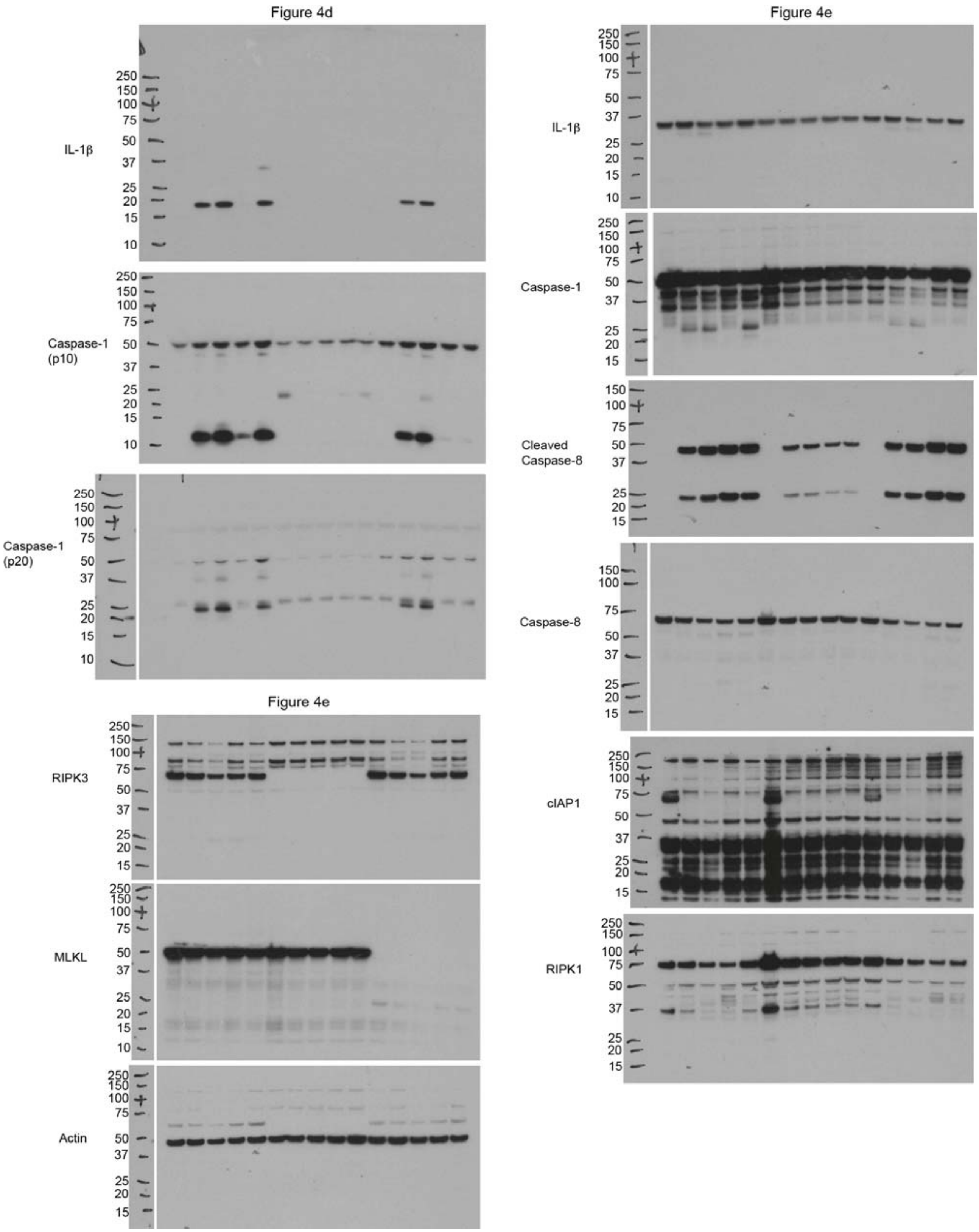

Supplementary Figure 12.
Whole gel scans of immunoblots presented in Figure 4d and 4e.

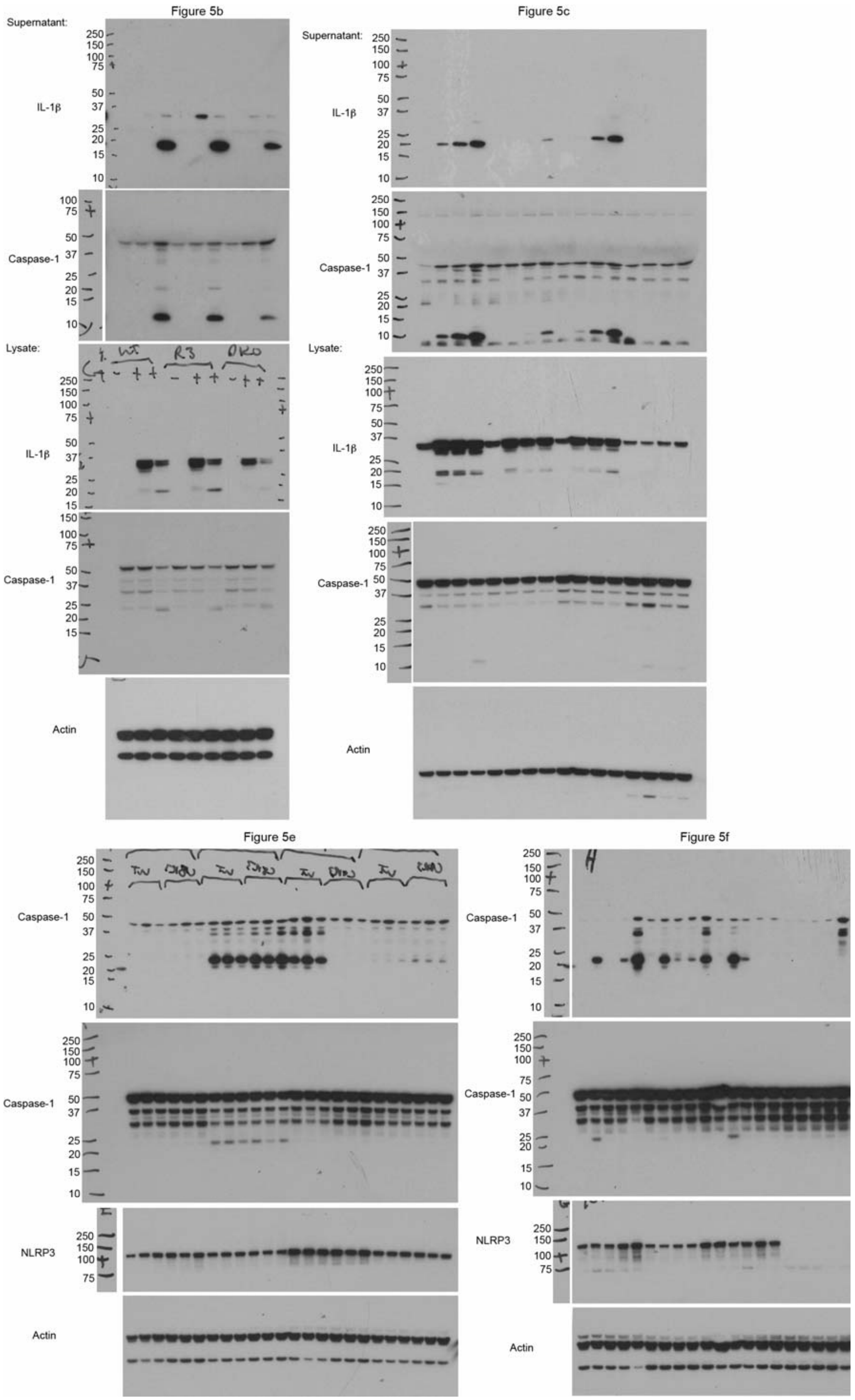

Supplementary Figure 13.
Whole gel scans of immunoblots presented in Figure 5b, 5c, 5e and 5f.

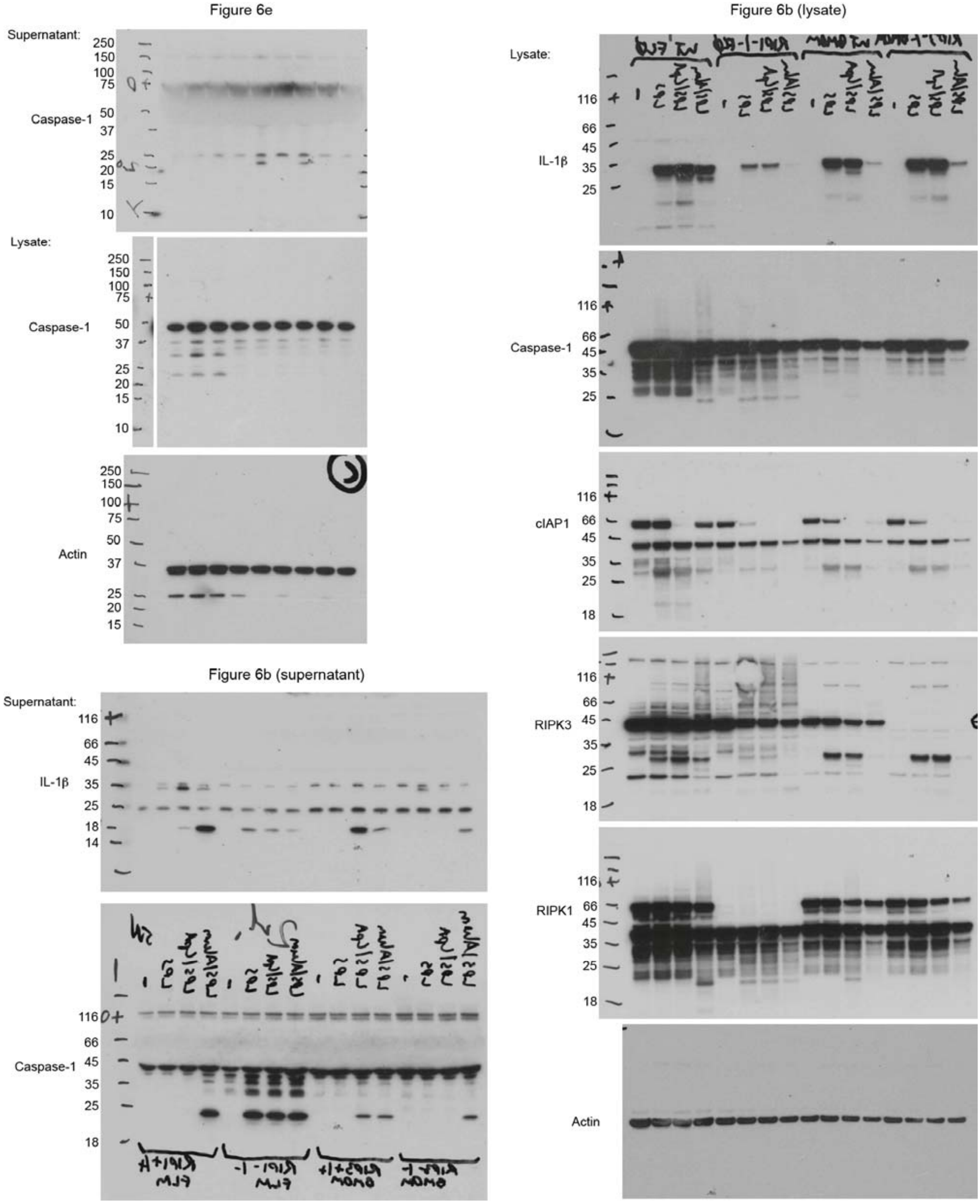

Supplementary Figure 14.
Whole gel scans of immunoblots presented in Figure 6b and 6e.

Supplementary Methods

Mice

Conditional cIAP1 (*Birc2*$^{flox/flox}$) and cIAP2 (*Birc3*$^{FRT/FRT}$) mice produced on a C57BL/6 background [1], and *Xiap*$^{-/-}$ mice [2] backcrossed onto a C57BL/6 background for >10 generations. Ubiquitous inactivation of cIAP2 expression was achieved by FlpE transgene (B6;SJL-Tg:F4(ACTFLPe)9205Dym/WEHI). These mice were utilized to generate mice with conditional deletion of cIAP1 in myeloid cells (Lysozyme M Cre transgenic); *cIAP1*^{LysMcre}*cIAP2*$^{-/-}$ and *cIAP1*^{LysMcre}*XIAP*$^{-/-}$*cIAP2*$^{-/-}$, as previously described [3]. Alternatively, tamoxifen inducible deletion of cIAP1 was achieved using the Cre-ERT2 transgenic mice [4]; *cIAP1*^{ERcre}*XIAP*$^{-/-}$*cIAP2*$^{-/-}$. All mutant mice, *cIAP1*$^{-/-}$, *cIAP2*$^{-/-}$, *Xiap*$^{-/-}$, *Xiap*$^{-/-}$*cIAP2*$^{-/-}$, caspase-8$^{flox/flox}$ [5], *Ripk1*$^{-/-}$ [6], *Ripk3*$^{-/-}$ [7], *Mlkl*$^{-/-}$ [8], *Ripk3*$^{-/-}$*Caspase-8*$^{-/-}$ and *Ripk1*$^{-/-}$*Ripk3*$^{-/-}$ [9], *Nlrp3*$^{-/-}$ [10], caspase-*1*$^{-/-}$ (also deficient in caspase-11)[11], *Tnf*$^{-/-}$[12], *IL-1R*$^{-/-}$[13], *IL-1α*$^{-/-}$[14], *Myd88*$^{-/-}$[15] and *Trif*$^{-/-}$ [16] mice were either generated or backcrossed onto a C57BL/6 background for 8-10 generations. *Caspase-8*$^{flox/flox}$ mice were also crossed with Lysozyme M Cre (myeloid specific) transgenic mice[17]. C57BL/6 (Ly5.2) and B6.SJL-*Ptprc*^{a} (C57BL/6 CD45.1-congenic; Ly5.1) mice were purchased from WEHI Bioservices Animal supplies (Kew, Australia).

IAP antagonist compounds

General. ^{1}H and ^{13}C NMR spectra were obtained at 300 and 75 MHz, respectively, using a Varian L600 spectrometer using tetramethylsilane as the internal standard. For all proline-containing analogs where two sets of resonances are observed owing to the presence of conformational isomers, the signal set for the major conformational isomer is reported. Reactions were routinely performed under a nitrogen atmosphere using standard glassware and high purity, commercial-grade solvents. Amino acid derivatives were purchased from Bachem, Fluka, Chem-Impex or other reputable suppliers and used without further purification unless stated otherwise. Unless indicated, amino acids and derivatives have the L-configuration. LC/MS analysis was performed on a Thermo-Fisher MSQ Plus instrument with a Gemini 5 μ C6-Phenyl 110 Å column (50 × 4.60 mm) using standard gradient conditions (A: water containing 0.1% HOAc v/v; B: acetonitrile containing 0.1% HOAc v/v); MS data were acquired with ESI, positive ionization; UV detection at 254 nm. Compound purifications were performed using flash silica gel chromatography or by preparative high performance liquid chromatography using a Varian Prostar system either in normal phase (SiO_2, EtOAc/hexane, 250 × 41.4 mm) or reverse phase (C18, 100 Å, 60 μ, 250 × 41.4 mm). Abbreviations: Boc: *tert*-butoxycarbonyl; TEA: triethylamine; DMAP: 4-dimethylaminopyridine; DCM: dichloromethane; Cbz: benzyloxycarbonyl; DIPEA: diisopropylethylamine; HPLC: high performance liquid chromatography; MHz: megahertz; MTBE: methyl *tert*-butyl ether; NMM: N-methylmorpholine; NMP: N-methylpyrrolidinone; HATU: *O*-(7-azabenzotriazol-1-yl)-*N*,*N*,*N′*,*N′*-tetramethyluronium hexafluorophosphate; TFA: trifluoroacetic acid; THF: tetrahydrofuran; EtOAc: ethyl acetate; HOAc: acetic acid; ACN: acetonitrile; Ac_2O: acetic anhydride.

The Preparation of GT13031 (designated 031 in main manuscript):

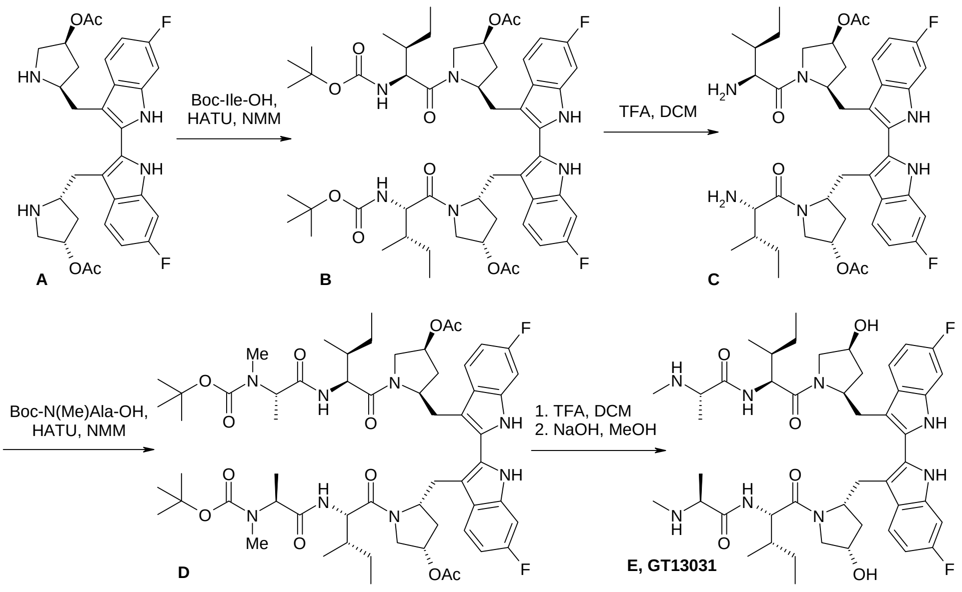

A representative procedure for the preparation of **E, GT13031** is provided below [18]:

Acetic acid 5-{3'-[4-acetoxy-1-(2-tert-butoxycarbonylamino-3-methyl-pentanoyl)-pyrrolidin-2-ylmethyl]-6,6'-difluoro-1H,1'H-[2,2']biindolyl-3-ylmethyl}-1-(2-tert-butoxycarbonylamino-3-methyl-pentanoyl)-pyrrolidin-3-yl ester (B): To a solution containing Boc-Ile-OH (100 mmol) and HATU (110 mmol) in anhydrous NMP (150 mL) at 0 ºC was added NMM (150 mmol) followed by a solution of **A** [1] (42 mmol) in NMP (100 mL). The reaction mixture was slowly warmed to ambient temperature. After 16 h, the reaction mixture was diluted with MTBE (1000 mL) and the heterogeneous mixture was washed with water (500 mL). The layers were separated and the organic phase formed a heterogeneous suspension. MTBE (1000 mL) and EtOAc (500 mL) were added and the now-homogeneous solution was washed successively with 1 N HCl (2 × 100 mL), saturated aqueous $NaHCO_3$ (2 × 100 mL), brine, dried over anhydrous Na_2SO_4, filtered, and concentrated. The residue was dissolved in 1:1 DCM/MeOH (600 mL) and DCM (ca. 200 mL) was removed via distillation at 50 °C. MeOH (200 mL) was added and additional solvent was removed (ca. 200 mL) at 50 °C. The heterogeneous mixture was cooled at -5 °C. After 16 h, the solid was collected by vacuum filtration and washed with cold MeOH. The solid was dried under high vacuum to afford **B**. Mass spectrum (ESI), *m/z* 977.4 [(M+H)+; calcd for $C_{52}H_{70}F_2N_6O_{10}$: 976.51].

Acetic acid 5-{3'-[4-acetoxy-1-(2-amino-3-methyl-pentanoyl)-pyrrolidin-2-ylmethyl]-6,6'-difluoro-1H,1'H-[2,2']biindolyl-3-ylmethyl}-1-(2-amino-3-methyl-pentanoyl)-pyrrolidin-3-yl ester (C): A solution containing **B** (30 mmol) in DCM (200 mL) was cooled to 0 ºC. TFA (50 mL) was added and the reaction was monitored by LC/MS analysis until complete conversion of **B** to **C** (ca. 3 h). The solvent was removed *in vacuo* and the residue was dissolved in EtOAc (ca. 1 L). The EtOAc solution was carefully poured into a saturated aqueous $NaHCO_3$/ice/water mixture to neutralize the residual TFA. The organic phase was separated and washed twice with saturated aqueous $NaHCO_3$ then once with brine. The combined aqueous washes were back-extracted with EtOAc (2 × 100 mL) and the combined organic extracts were dried over anhydrous Na_2SO_4, filtered, and concentrated to afford crude **C**. Mass spectrum (ESI), *m/z* 777.3 [(M+H)+; calcd for $C_{42}H_{54}F_2N_6O_6$: 776.41].

Acetic acid 5-{3'-[4-acetoxy-1-[2-(2-methyl-(tert-butoxycarbonyl)-amino-propionylamino)-pentanoyl]-pyrrolidin-2-ylmethyl]-6,6'-difluoro-1H,1'H-[2,2']biindolyl-3-ylmethyl}-1-[2-(2-methyl-(tert-butoxycarbonyl)-amino-propionylamino)-pentanoyl]-pyrrolidin-3-yl ester (D): To a solution containing Boc-N(Me)Ala-OH (72 mmol) and HATU (80 mmol) in anhydrous NMP

(150 mL) at 0 ºC was added NMM (105 mmol) followed by addition of **C** (30 mmol) in NMP (200 mL). The resulting mixture was allowed to warm to ambient temperature. After 16 h, the reaction mixture was diluted with diethyl ether (1 L) and washed successively with water (1 L), 1 N HCl (2 × 100 mL), saturated aqueous $NaHCO_3$ (2 × 100 mL), brine, dried over anhydrous Na_2SO_4, filtered, concentrated to afford crude **D**. Mass spectrum (ESI), *m/z* 1147.7 [(M+H)+; calcd for $C_{60}H_{84}F_2N_8O_{12}$: 1146.62].

***N*-{1-[2-(6,6'-Difluoro-3'-{4-hydroxy-1-[3-methyl-2-(2-methylamino-propionylamino)-pentanoyl]-pyrrolidin-2-ylmethyl}-1H,1'H-[2,2']biindolyl-3-ylmethyl)-4-hydroxy-pyrrolidine-1-carbonyl]-2-methyl-butyl}-2-methylamino-propionamide (E):** A solution containing **D** (26 mmol) in DCM (150 mL) was cooled to 0 ºC. TFA (50 mL) was added. After 30 min, the reaction mixture was warmed to ambient temperature and monitored until LC/MS analysis revealed complete consumption of **D**. The solvent was removed *in vacuo* and the residue was dissolved in EtOAc (500 mL) and *carefully* poured onto an aqueous $NaHCO_3$/ice mixture. The aqueous phase was separated and back-extracted with EtOAc (2 × 250 mL). The combined organic extracts were washed several times with saturated aqueous $NaHCO_3$, and then brine, dried over anhydrous Na_2SO_4, filtered, and concentrated to afford the crude intermediate diamine which was used without further purification. Mass spectrum (ESI), *m/z* 947.5 [(M+H)+; calcd for $C_{50}H_{68}F_2N_8O_8$: 946.51].

To a solution containing the crude intermediate diamine in MeOH (200 mL) was added 1 M NaOH (80 mL) at 0 ºC. The reaction mixture was degassed and maintained under a nitrogen atmosphere wrapped with aluminum foil. The ice-bath was removed. After 60 min, the MeOH was removed *in vacuo* and the residue was diluted with water (200 mL) and extracted with EtOAc (500 mL). The aqueous phase was separated and back-extracted with EtOAc (2 × 150 mL). The combined organic extracts were washed with brine and dried over anhydrous Na_2SO_4, filtered, and concentrated to afford crude **E**.

The crude **E** was dissolved in MeOH (50 mL) and EtOAc (200 mL). The volume was reduced (50%) by distillation at reduced pressure at 60 ºC using a rotary evaporator. MTBE (300 mL) was added and the cloudy solution was warmed to 60 ºC. After 30 min, the solution was cooled to ambient temperature and then maintained at -5 ºC.

After 16 h, the solid was collected by vacuum filtration and washed with cold 25% EtOAc/MTBE and dried under high vacuum at ambient temperature to afford **E** (**GT13031**). ^{1}H NMR (DMSO-d_6, 300 MHz): δ11.94 (s, 2H), 8.71 (d, *J* = 7.8 Hz, 2H), 7.82 (dd, *J* = 8.7, 5.7 Hz, 2H), 7.45 (dd, *J* = 9.8, 2.4 Hz, 2H), 6.93 (m, 2H), 5.69 (br s, 2H), 4.43 (m, 4H), 4.22 (m, 2H), 3.93 (m, 2H), 3.76 (d, *J* = 11.1 Hz, 2H), 3.64 (q, *J* = 6.6 Hz, 2H), 3.40 – 3.55 (m, 4H), 2.48 (m, 4H), 2.44 (s, 6H), 1.74 – 2.06 (m, 6H), 1.64 (m, 2H), 1.34 (d, *J* = 6.9 Hz, 6H), 1.26 (m, 2H) 1.02 (d, *J* = 6.9 Hz, 6H), 0.91 (t, *J* = 7.2 Hz, 6H) ppm; ^{13}C NMR (DMSO-d_6, 75 MHz): δ172.3, 171.5, 161.6, 158.5, 137.3, 137.1, 128.3, 126.4, 120.8, 109.4, 108.7,108.4, 98.4, 98.1, 70.7, 59.9, 57.7, 57.0, 55.6, 36.8, 36.5, 32.7, 28.2, 25.1, 17.5, 15.7, 11.5 ppm; Mass spectrum (ESI) *m/z* 863.3 [(M+H)+; calcd for $C_{46}H_{64}F_2N_8O_6$: 862.49].

The Preparation of GT14883 (designated 883 in main manuscript):

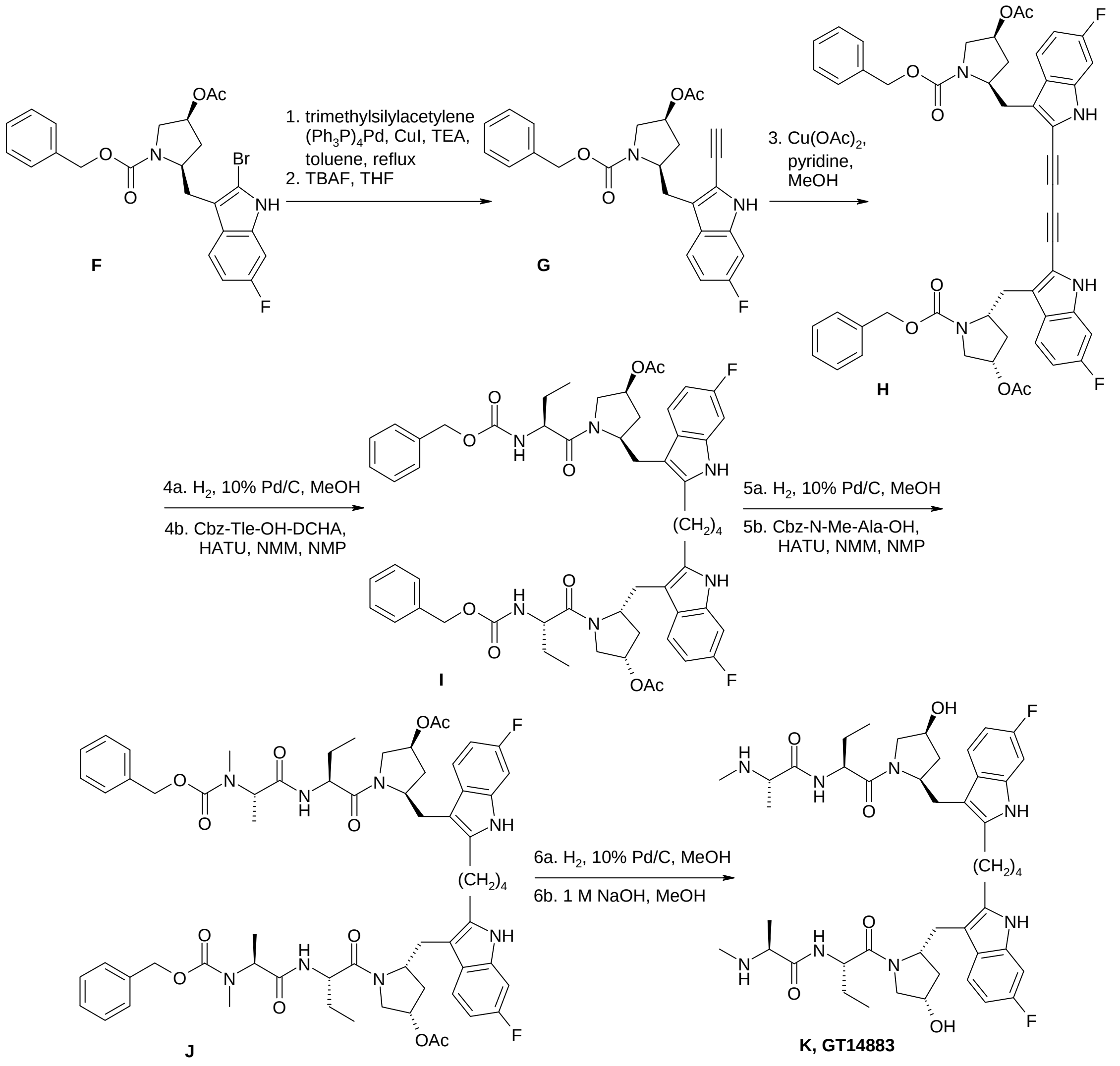

4*S*-Acetoxy-2*R*-(2-ethynyl-6-fluoro-1H-indol-3-ylmethyl)-pyrrolidine-1-carboxylic acid benzyl ester (**G**): To a degassed solution of bromide **F** [19] (1.5 g, 3.07 mmol) in toluene (20 mL) was added $(Ph_3P)_4Pd(0)$ (358 mg, 0.31 mmol), CuI (59 mg, 0.31 mmol), trimethlsilylacetylene (3.04 g, 30.9 mmol), and TEA (1.55 g, 15.4 mmol). The reaction was immersed into a pre-heated (100 °C) oil bath. After 3 h, the reaction mixture was cooled and diluted with EtOAc, washed successively with water, 1 M HCl, saturated aqueous $NaHCO_3$, and brine; dried over anhydrous Na_2SO_4, filtered, and concentrated. The residue was purified by normal-phase preparative HPLC (2” Dynamax SiO_2 column; Flow: 40 mL/min; Method: 10-50% EtOAc/hexane over 30 min) to afford 470 mg of the intermediate TMS-acetylene (30%). ^{1}H NMR (300 MHz, solvent): δ 7.78 (m, 1H), 7.54 (t, *J* = 12.9 Hz, 2H), 7.11 (m, 8H), 6.97 (t, *J* = 6.9 Hz, 4H), 6.61 (m, 2H), 6.48 (s, 1H), 4.98 (m, 4H), 4.02 (m, 2H), 3.56 (m, 2H), 3.44 (q, *J* = 11.7 Hz, 2H), 3.26 (t, *J* = 13.5 Hz, 2H), 3.03 (t, *J* = 12.0 Hz, 2H), 2.82 (t, *J* = 11.7 Hz, 1H), 1.85 (m, 4H), 1.70 (m, 2H), 0.31 (d, *j* = 5.4 Hz, 6H), 0.02 (m, 9H) ppm. Mass spectrum (ESI) *m/z* 506.8 [(M)+; calcd for $C_{28}H_{31}FN_2O_4Si$: 506.20].

The intermediate TMS-acetylene (470 mg) was dissolved in THF (10 mL) and cooled to 0 °C. Tetra(*n*-butyl)ammonium fluoride (1 M/THF, 1.39 mL) was added and the reaction mixture was stirred for 3 h at 0 °C. The reaction mixture was diluted with EtOAc, washed successively with water, saturated aqueous NH_4Cl, water, and brine; dried over anhydrous Na_2SO_4, filtered, and concentrated. The residue was purified by normal-phase preparative HPLC (2” Dynamax SiO_2 column; Flow: 40 mL/min; Method: 20-100% EtOAc/hexane over 30 min) to afford 290 mg of **G** (72%). ^{1}H NMR (300 MHz, solvent): δ8.19 (d, *J* = 5.1 Hz, 2H), 7.84 (t, *J* = 5.4 Hz, 1H), 7.40 (m,

8H), 7.08 (dd, *J* = 5.7, 7.8 Hz, 1H), 6.93 (q, *J* = 9.6 Hz, 2H), 6.56 (t, *J* = 9 Hz, 1H), 5.22 (m, 6H), 4.32 (m, 1H), 4.20 (m, 1H), 3.79 (m, 2H), 3.67 (q, *J* = 13.5 Hz, 2H), 3.52 (d, *J* = 14.1 Hz, 1H), 3.42 (s, 1H), 3.27 (d, *J* = 18.9 Hz, 2H), 3.09 (t, *J* = 12.0 Hz, 2H), 2.14 (m, 6H), 1.66 (s, 2H) ppm. Mass spectrum (APCI) *m/z* 435.2 [(M+H)+; calcd for $C_{25}H_{24}FN_2O_4$: 435.17].

4-Acetoxy-2-(2-{4-[3-(4-acetoxy-1-benzyloxycarbonyl-pyrrolidin-2-ylmethyl)-6-fluoro-1H-indol-2-yl]-buta-1,3-diynyl}-6-fluoro-1H-indol-3-ylmethyl)-pyrrolidine-1-carboxylic acid benzyl ester (**H**): A solution containing **G** (280 mg, 0.64 mmol), $Cu(OAc)_2$ (232 mg, 1.28 mmol) in pyridine (3 mL) and MeOH (3 mL) was stirred for 19 h at ambient temperature. The reaction mixture was concentrated to dryness; dissolved in EtOAc and washed successively with 1 M HCl (3×), saturated aqueous $NaHCO_3$, and brine; dried with anhydrous Na_2SO_4, filtered and concentrated. The residue was purified by normal-phase preparative HPLC (2” Dynamax SiO_2 column; Flow: 40 mL/min; Method: 30-100% EtOAc/hexane over 30 min) to afford 160 mg of **H** (57%). ^{1}H NMR (300 MHz, solvent): δ8.72 (s, 2H), 7.86 (dd, *J* = 6.3, 8.4 Hz, 1H), 7.4 (m, 9H), 7.05 (dd, *J* = 5.1, 8.4 Hz, 1H), 6.93 (t, *J* = 10.2 Hz, 2H), 6.54 (t, *J* = 9.3 Hz, 1H), 5.40 (m, 2H), 5.22 (dq, *J* = 6.6, 13.8 Hz, 4H), 4.27 (m, 1H), 4.16 (m, 1H), 3.72 (m, 4H), 3.54 (d, *J* = 12.6 Hz, 1H), 3.33 (d, *J* = 11.1 Hz, 1H), 3.11 (d, *J* = 11.4 Hz, 2H), 2.18 (s, 6H), 2.05 (m, 4H) ppm. Mass spectrum (APCI) *m/z* 867.4 [(M+H)+; calcd for $C_{50}H_{46}F_2N_4O_8$: 867.32].

Acetic acid 5-[2-(4-{3-[4-acetoxy-1-(2-benzyloxycarbonylamino-3,3-dimethyl-butyryl)-pyrrolidin-2-ylmethyl]-6-fluoro-1H-indol-2-yl}-butyl)-6-fluoro-1H-indol-3-ylmethyl]-1-(2-benzyloxycarbonylamino-3,3-dimethyl-butyryl)-pyrrolidin-3-yl ester (I): A solution containing **H** (420 mg, 0.48 mmol) and 10% Pd/C (100 mg) in MeOH (10 mL) was placed under an atmosphere of hydrogen. After 4 h, the reaction mixture was filtered and concentrated to provide 240 mg of crude bis-pyrrolidine (ca. 82%) which was used without further purification.

To a solution containing the crude bis-pyrrolidine (120 mg, 0.20 mmol), Cbz-Tle-OH-DCHA (197 mg, 0.44 mmol), and DIPEA (139 mg, 0.80 mmol) in NMP (3 mL) was added HATU (167 mg, 0.44 mmol) at ambient temperature. After 6 h, the reaction mixture was diluted with 1 M HCl and extracted with EtOAc (3×). The combined organic extracts were washed successively with 1 M HCl, saturated aqueous $NaHCO_3$, brine; dried over anhydrous Na_2SO_4, filtered and concentrated to afford 280 mg of **I** (quant.) as a brown solid which was taken forward without further purification. Mass spectrum (APCI) *m/z* 1101.63 [(M+H)+; calcd for $C_{62}H_{74}F_2N_6O_{10}$: 1100.54].

Acetic acid 5-[2-(4-{3-[4-acetoxy-1-(2-[benzyloxycarbonyl-methyl-amino]-propionylamino) -3,3-dimethyl-butyryl)-pyrrolidin-2-ylmethyl]-6-fluoro-1H-indol-2-yl}-butyl)-6-fluoro-1H-indol-3-ylmethyl]-1-(2--[benzyloxycarbonyl-methyl-amino]-propionylamino) -3,3-dimethyl-butyryl)-pyrrolidin-3-yl ester (J): A solution containing **I** (220 mg, 0.20 mmol) and 10% Pd/C (50 mg) in MeOH (10 mL) was placed under an atmosphere of hydrogen. After 19 h, the reaction mixture was filtered and concentrated to provide 210 mg of crude bis-amine (quant.) which was used without further purification.

To a solution containing the crude bis-amine (167 mg, 0.20 mmol), Cbz-*N*-Me-Ala-OH (104 mg, 0.44 mmol), and DIPEA (139 mg, 0.80 mmol) in NMP (3 mL) was added HATU (167 mg, 0.44 mmol) at ambient temperature. After 3 h, the reaction mixture was diluted with 1 M HCl and extracted with EtOAc (3×). The combined organic extracts were washed successively with 1 M HCl, saturated aqueous $NaHCO_3$, brine; dried over anhydrous Na_2SO_4, filtered and concentrated. The resultant residue was purified by normal-phase preparative HPLC (2” Dynamax SiO_2 column; Flow: 40 mL/min; Method: 30-100% EtOAc/hexane over 30 min) to afford 100 mg of **J** (39%) as an off-white solid. Mass spectrum (APCI) *m/z* 1293.63 [(M+Na)+; calcd for $C_{70}H_{88}F_2N_8O_{12}$: 1270.65].

5-[2-(4-{3-[4-hydroxy-1-(2-[methyl-amino]-propionylamino) -3,3-dimethyl-butyryl)-pyrrolidin-2-ylmethyl]-6-fluoro-1H-indol-2-yl}-butyl)-6-fluoro-1H-indol-3-ylmethyl]-1-(2--[methyl-amino]-propionylamino) -3,3-dimethyl-butyryl)-pyrrolidine (K, GT14883): A solution containing **J** (100 mg, 0.08 mmol) and 10% Pd/C (30 mg) in MeOH (3 mL) was placed under an atmosphere of hydrogen. After 19 h, the reaction mixture was filtered and concentrated to provide the crude bis-amine (quant.) which was used without further purification.

To a solution of the crude bis-amine in MeOH (3 mL) was added 1 M NaOH (500 μL). After 2 h the mixture was neutralized with HOAc and concentrated. The residue was purified by reverse-phase preparative HPLC (2" Dynamax C18 column; Flow: 40 mL/min; Method: 40-100% ACN/water containing 0.1% v/v HOAc over 40 min) to afford 46 mg of **K** (**GT14883**, 63%). ^{1}H NMR ($CDCl_3$ + d_4-MeOH, 300 MHz): δ7.70 (dd, J = 3.3, 5.1 Hz, 2H), 6.94 (t, J = 8.4 Hz, 2H), 6.80 (t, J = 9.0 Hz, 2H), 4.62 (m, 1H), 4.42 (m, 3H), 4.20 (m, 1H), 3.98 (m, 1H), 3.58 (m, 1H), 3.40 – 3.20 (m, 4H), 2.62 (s, 3H), 2.88 (m, 3H), 2.26 (m, 4H), 2.05 – 4.88 (m, 4H), 1.78 (m, 8H), 1.32 – 1.21 (m, 6H), 1.03 – 0.88 (m, 6H) ppm; ^{13}C NMR ($CDCl_3$ + d_4-MeOH, 75 MHz): δ175.4, 171.1, 170.6, 161.1, 158.0, 137.2, 135.7, 125.6, 124.6, 119.6, 118.1, 108.0, 107.5, 107.2, 96.9, 96.6, 70.7, 69.0, 68.4, 67.3, 65.9, 64.5, 59.9, 59.5, 55.8, 51.9, 42.2, 41.5, 39.9, 36.7, 34.7, 30.4, 29.2, 28.2, 27.4, 26.8, 25.9, 25.7, 19.3, 11.4, 9.9 ppm; Mass spectrum (ESI) m/z 919.5 [(M)+; calcd for $C_{50}H_{72}F_2N_8O_6$: 918.55].

Supplementary References

1. Gardam S, Turner VM, Anderton H, Limaye S, Basten A, et al. (2011) Deletion of cIAP1 and cIAP2 in murine B lymphocytes constitutively activates cell survival pathways and inactivates the germinal center response. Blood 117: 4041-4051.
2. Harlin H, Reffey SB, Duckett CS, Lindsten T, Thompson CB (2001) Characterization of XIAP-deficient mice. Mol Cell Biol 21: 3604-3608.
3. Wong W, E. VJ, Lalaoui N, Chau D, Bankovacki A, et al. (2014) cIAPs and XIAP regulate myelopoiesis through cytokine production in a RIPK1 and RIPK3 dependent manner. Blood 123: 2562-2572.
4. Seibler J, Zevnik B, Kuter-Luks B, Andreas S, Kern H, et al. (2003) Rapid generation of inducible mouse mutants. Nucleic Acids Res 31: e12.
5. Beisner DR, Ch'en IL, Kolla RV, Hoffmann A, Hedrick SM (2005) Cutting edge: innate immunity conferred by B cells is regulated by caspase-8. J Immunol 175: 3469-3473.
6. Kelliher MA, Grimm S, Ishida Y, Kuo F, Stanger BZ, et al. (1998) The death domain kinase RIP mediates the TNF-induced NF-kappaB signal. Immunity 8: 297-303.
7. Newton K, Sun X, Dixit VM (2004) Kinase RIP3 is dispensable for normal NF-kappa Bs, signaling by the B-cell and T-cell receptors, tumor necrosis factor receptor 1, and Toll-like receptors 2 and 4. Mol Cell Biol 24: 1464-1469.
8. Murphy JM, Czabotar PE, Hildebrand JM, Lucet IS, Zhang JG, et al. (2013) The Pseudokinase MLKL Mediates Necroptosis via a Molecular Switch Mechanism. Immunity 39: 443-453.
9. Rickard JA, O'Donnell JA, Evans JM, Lalaoui N, Poh AR, et al. (2014) RIPK1 Regulates RIPK3-MLKL-Driven Systemic Inflammation and Emergency Hematopoiesis. Cell 157: 1175-1188.
10. Brydges SD, Mueller JL, McGeough MD, Pena CA, Misaghi A, et al. (2009) Inflammasome-mediated disease animal models reveal roles for innate but not adaptive immunity. Immunity 30: 875-887.
11. Kuida K, Lippke JA, Ku G, Harding MW, Livingston DJ, et al. (1995) Altered cytokine export and apoptosis in mice deficient in interleukin-1 beta converting enzyme. Science 267: 2000-2003.
12. Korner H, Cook M, Riminton DS, Lemckert FA, Hoek RM, et al. (1997) Distinct roles for lymphotoxin-alpha and tumor necrosis factor in organogenesis and spatial organization of lymphoid tissue. Eur J Immunol 27: 2600-2609.
13. Thomas HE, Irawaty W, Darwiche R, Brodnicki TC, Santamaria P, et al. (2004) IL-1 receptor deficiency slows progression to diabetes in the NOD mouse. Diabetes 53: 113-121.
14. Horai R, Asano M, Sudo K, Kanuka H, Suzuki M, et al. (1998) Production of mice deficient in genes for interleukin (IL)-1alpha, IL-1beta, IL-1alpha/beta, and IL-1 receptor antagonist shows that IL-1beta is crucial in turpentine-induced fever development and glucocorticoid secretion. J Exp Med 187: 1463-1475.

15. Adachi O, Kawai T, Takeda K, Matsumoto M, Tsutsui H, et al. (1998) Targeted disruption of the MyD88 gene results in loss of IL-1- and IL-18-mediated function. Immunity 9: 143-150.
16. Yamamoto M, Sato S, Hemmi H, Hoshino K, Kaisho T, et al. (2003) Role of adaptor TRIF in the MyD88-independent toll-like receptor signaling pathway. Science 301: 640-643.
17. Clausen BE, Burkhardt C, Reith W, Renkawitz R, Forster I (1999) Conditional gene targeting in macrophages and granulocytes using LysMcre mice. Transgenic Res 8: 265-277.
18. Condon SM, LaPorte MG, Deng Y, Rippin, S, "SMAC Mimetic," US 8,603,816 (2013).
19. Condon SM, LaPorte MG, Deng Y, Rippin, S, "Dimeric IAP Inhibitors," US 7,517,906 (2009).
